# Prevalence of Mental Disorders in the WHO Eastern Mediterranean Region: A Systematic Review and Meta-Analysis

**DOI:** 10.3389/fpsyt.2021.665019

**Published:** 2021-07-14

**Authors:** Alina Zuberi, Ahmed Waqas, Sadiq Naveed, Md Mahbub Hossain, Atif Rahman, Khalid Saeed, Daniela C. Fuhr

**Affiliations:** ^1^Department of Health Services Research and Policy, London School of Hygiene and Tropical Medicine, London, United Kingdom; ^2^Department of Primary Care and Mental Health, Institute of Population Health Sciences, University of Liverpool, Liverpool, United Kingdom; ^3^Department of Child Psychiatry, Institute of Living, Hartford, CT, United States; ^4^School of Public Health, Texas A&M University, College Station, TX, United States; ^5^World Health Organization, Regional Office for the Eastern Mediterranean, Cairo, Egypt

**Keywords:** prevalence, mental disorders, Eastern Mediterranean, meta-analyses, systematic review & meta-analysis, conflict

## Abstract

**Objectives:** To synthesize the prevalence of mental and substance use disorders in countries of the Eastern Mediterranean Region (EMR) of the World Health Organization.

**Methods:** The literature search was conducted across several databases in two phases. First, we searched for systematic reviews and/or meta-analyses published before 2014, reporting prevalence estimates for mental disorders in the EMR. Then, we identified new primary cross-sectional or longitudinal studies published between 2014 and 2020. Studies were included if they had a sample size of ≥ 450 and were conducted among the general adult population. Current, period and lifetime prevalence estimates for each disorder were pooled using random-effects meta-analyses, and subgroup analyses and meta-regressions were conducted.

**Findings:** Prevalence estimates were extracted from 54 cross-sectional studies across 15 countries within the EMR. Pooled analyses of current, period and lifetime prevalence showed the highest prevalence for depression (14.8%, 95% confidence interval, CI: 10.7–20.1%), followed by generalized anxiety disorder (GAD) (10.4%, 95% CI: 7.1–14.7%), post-traumatic stress disorder (7.2%, 95% CI: 2.9–16.6%), substance use (4.0%, 95% CI: 3.1–5.2%), obsessive compulsive disorder (2.8%, 95% CI: 1.6–4.9%), phobic disorders (1.8%, 95% CI: 1.1–2.8%), panic disorders (1.1%, 95% CI: 0.6–2.2%), bipolar disorders (0.7%, 95% CI: 0.3–1.6%), and psychosis (0.5%, 95% CI: 0.3–0.9%). Populations exposed to adverse events had higher prevalence of mental disorders than the general population. Period and lifetime prevalence showed little difference across mental disorders. More pronounced differences in prevalence were seen for depression and GAD, specifically between current and lifetime prevalence (depression: current prevalence 20.5% (95% CI: 14.9–27.4%), vs. lifetime prevalence: 4.2% (95%CI: 1.8–9.6%); GAD: current prevalence 10.3% (95% CI: 6.1–17.0), vs. lifetime prevalence: 4.5% (95% CI: 2.4–8.3%). Differences between current and lifetime prevalence of mental disorders may be due to the use of different screening instruments and thresholds being applied.

**Conclusion:** The prevalence of mental and substance use disorders in the EMR is high. Despite substantial inter-survey heterogeneity, our estimates align with previous global and regional data on mental disorders. Our meta-review provides new evidence on the burden of mental health problems in the EMR.

**Systematic Review Registration:** PROSPERO, https://www.crd.york.ac.uk/prospero/display_record.php?ID=CRD42020187388.

## Introduction

Mental disorders contribute significantly to the global burden of disease, with common mental disorders (CMDs) such as depression, anxiety and post-traumatic stress disorders (PTSD) accounting for 41.9% of the burden (1). Globally, the 12-month prevalence of CMDs in adults is estimated to be 17.6% and the lifetime prevalence is 29.2% (2). Research suggests that prevalence of mental disorders is increasing in low-and-middle-income countries due to population growth and aging (3). An extensive body of research suggests that the experience of war, conflict, population displacement, infrastructure damage and unemployment leads to increased symptoms of depression, anxiety, trauma and stress-related disorders (4), and this is evident in countries located in the Eastern Mediterranean Region (EMR) of the World Health Organization (WHO) in which the 12-month prevalence of CMDs ranges between 11 and 40.1% (5) (list of EMR member countries included in Annex 1). Almost 85% of the EMR population have experienced a humanitarian crisis within the past two decades (6), with a higher prevalence of psychological distress seen in EMR populations experiencing significant conflict (5).

Previous research investigated the prevalence of specific mental disorders in the EMR region (7, 8) or within single EMR countries (9, 10). For example, Travers et al. synthesized evidence on the prevalence of major depressive disorder within Africa and the Middle East (7), and Naveed et al. reported on the pooled prevalence of CMDs and substance use disorders in South Asia (8). Additionally, Sadeghirad et al. reviewed the prevalence of major depressive disorder in Iran (9), and Mirza and Jenkins assessed the evidence on the prevalence of anxiety and depressive disorders in Pakistan (10). However, there is a lack of recently synthesized information on the overall prevalence and determinants of priority mental disorders within the EMR.

This systematic review and meta-analysis aims to provide updated pooled prevalence estimates of mental and substance use disorders within the EMR. The objectives of this review are to (a) aggregate prevalence estimates of mental and substance use disorders within the EMR (present pooled estimates of current, period and lifetime prevalence; and separate estimates for current, period and lifetime prevalence, respectively); (b) estimate the prevalence among populations exposed to adversity (such as refugees or victims of natural disasters); and (c) investigate socio-demographic and country correlates of mental and substance use disorders.

## Methods

This review follows the Preferred Reporting Items for Systematic reviews and Meta-Analysis (PRISMA) guidelines (PRISMA checklist included in Annex 2) (11). The protocol was registered in the PROSPERO database in July 2020 with the registration number: CRD42020187388.

### Inclusion and Exclusion Criteria

We included studies reporting current (such as 1- or 2-week, or 1 month), period (such as 6 or 12 months) or lifetime prevalence estimates of the following mental disorders—depressive disorders, bipolar disorders, generalized anxiety disorder (GAD), panic disorder, obsessive-compulsive disorder (OCD), social phobia, PTSD, acute stress disorder or any other anxiety disorder, psychosis or substance use (including alcohol-use disorders, or harmful use of or dependence on tobacco, cannabis, opioids, stimulants and non-prescriptions drugs). The population of interest was the general adult population aged 18 years or older. Exceptions were made for studies that included participants < 18 years of age, and these were included if the majority of the sample (>70%) were 18+ years or the reported mean age was > 18+ years. Studies were included if they had a sample size ≥ 450. This criterion has been used previously and ensures adequate statistical power to provide precise, reliable and stable prevalence estimates of the general population (2, 12, 13). Annex 3 provides further information about our inclusion and exclusion criteria.

### Literature Search

Previous reviews published until the end of 2013, which included data on the EMR, were used to identify primary cross-sectional and longitudinal studies that met the above-outlined eligibility criteria (7, 10, 14–16). Moreover, experts in the field were consulted to retrieve potential reviews that met the current review's purpose (2, 8). The literature search was conducted in five electronic databases, namely CINAHL Plus, PsycINFO, PubMed, Scopus and Web of Science. The search strategy was developed in PubMed, and Table 1 presents the specific keywords used. Annex 4 provides the exact search queries. These searches were amended in other databases to obtain optimal searching; for example, (.ti) and (.ti.ab) textwords were used in PsycINFO, instead of [Title] and [Title/Abstract] in PubMed.

**Table 1:** Search terms.

	**Keywords**
Disorder type	psych^*^ OR “mental” OR “mood disorders” OR “Depression” OR “Depressive Disorder” OR “Substance abuse” OR “substance-use” OR “substance-related disorders” OR “post-traumatic stress disorder” OR “post-traumatic stress disorder” OR “PTSD” OR “obsessive compulsive disorder” OR “OCD” OR “bipolar disorder” OR “Anxiety” OR “Panic disorder” OR “schizophrenia” OR “GAD” OR “Acute stress disorder”
Outcomes	“Prevalence” OR “Frequency” OR “epidemiology” OR “epidemiological” OR “proportion” OR “cases” OR “Odds” OR “Risks” OR “Status” OR “Associated factor” OR “Distribution” OR “Determinants” OR “Risk factor” OR correlate^*^ OR predictor^*^
Study design	“Systematic Review” OR “meta-analysis” OR “pooled estimate” OR “pooled effect” OR “Systematic Literature Review” OR “Meta- regression” OR “meta-analytic”
	Cross-section^*^ OR “longitudinal” OR “prospective” OR cohort^*^
Region	“Global” OR “EMR” OR “EMRO” OR “Eastern Mediterranean Region” OR “Middle East” OR “Middle Eastern Countries” OR “Northern Africa” OR “North Africa” OR “South Asia” OR “Western Asia” OR “Afghanistan” OR “Bahrain” OR “Djibouti” OR “Egypt” OR “Iran” OR “Iraq” OR “Jordan” OR “Kuwait” OR “Lebanon” OR “Libya” OR “Morocco” OR “Oman” OR “Pakistan” OR “Palestine” OR “Qatar” OR “Saudi Arabia” OR “Somalia” OR “Sudan” OR “Syria” OR “Tunisia” OR “United Arab Emirates” OR “UAE” OR “Yemen”

Two searches were run on each database. The first search aimed to identify systematic reviews and meta-analyses published until the end of 2013. The second search focused on identifying new primary research such as cross-sectional and longitudinal studies conducted within the EMR countries from 2014 onwards to obtain recent prevalence estimates of mental disorders in the region that were not published in any of the previous systematic reviews (7, 10, 14–16). The literature search was conducted in July 2020 and restricted to English. We also repeated the search up to 2013 and did not find any non-included paper meeting inclusion criteria.

### Study Selection

Title and abstracts were screened by one author (AZ), and a second reviewer (MH) screened 40% (*n* = 4,921) of the articles. This was done in parallel to allow quality checks to be performed by analyzing inter-rater reliabilities. Any discrepancies were resolved by a third team member (SN). The full texts were reviewed by at least two authors (AZ, AW, SN, or MH) working independently from each other. Studies with overlapping samples (both time periods and regions) were considered as duplicates, with only the one which had complete data introduced to our meta-analyses.

### Data Extraction

Data were extracted by four authors (AZ, AW, SN, and MH) using a manually constructed data extraction form (information on the type of data extracted is included in Annex 5). Data from each study were extracted by at least two reviewers working independently from each other. In case of discrepancies, a senior reviewer (DF) was consulted.

### Data Analysis

The Comprehensive Meta-Analysis software (17) was used to calculate the pooled prevalence rates for mental and substance use disorders using random-effects by employing the DerSimonian and Laird variance estimator and logit transformation. A random-effects model was chosen due to the anticipated heterogeneity in the data. The extent of heterogeneity was assessed using several statistics, including the *I*^2^ considered significant at > 40% and substantial at > 75%, Cochran's Q-statistic considered significant at *p* < 0.05. Sensitivity analyses were conducted by running pooled analyses, with sequential removal of each study to assess its contribution to pooled prevalence rates and any significant changes therein. In addition, we assessed the contribution of outlier studies to quantify their contribution to pooled prevalence rates. Begg's funnel plot (18) and Egger's regression test (19) were used to evaluate publication bias, with significance set at *p* < 0.10. Publication bias was adjusted for using Duval & Tweedie's Trim and Fill method (20). Subgroup analyses were conducted for subgroups reported in more than four studies (8). This was to ensure appropriate statistical power to detect any differences. Furthermore, meta-regression analyses were conducted on the age of participants and country gross domestic product (GDP). Both of these are significant predictors of some of the mental disorders and substance use; for instance, mental well-being is consistently shown to be affected by a country's prosperity and poverty rates.

### Quality Assessment

The quality of studies was assessed using the Joanna Briggs Institute (JBI) Critical Appraisal Checklist (21) for prevalence studies by at least two reviewers (AZ, SN, or MH) working independently from each other, without blinding to the journal or authorship. The checklist consists of 8 items that assess the chance of bias in a study's design, conduct and data analysis. Further details about the item responses and the process of calculating the quality scores for each study are provided in Appendix 17.

## Results

**Figure 1:**
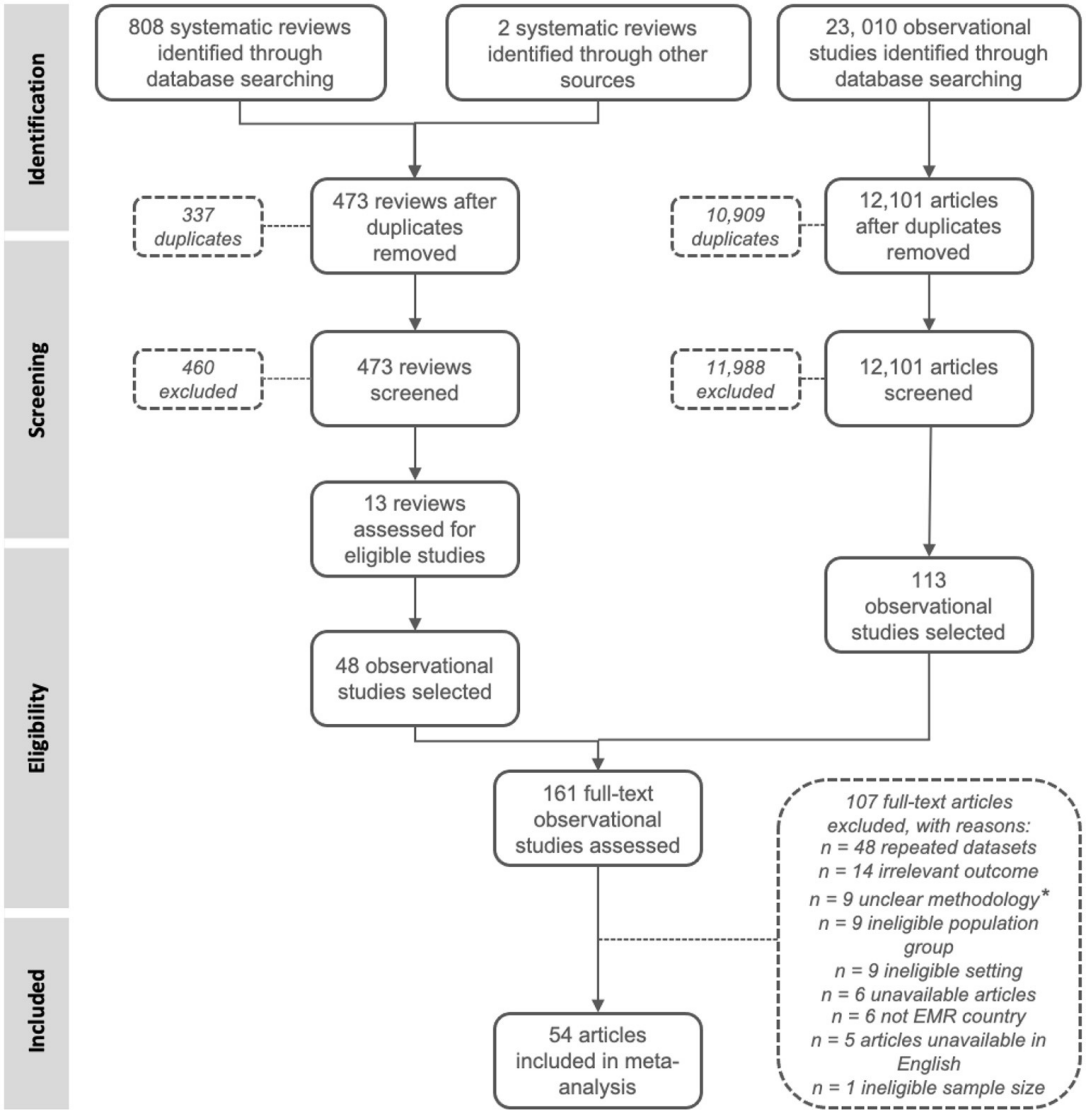
Flowchart. ^*^studies that weren't transparent about their methodologies, setting and time period.

The initial literature search yielded 808 systematic reviews (search #1) and 23,010 observational studies (search #2). Two additional systematic reviews were identified by experts (2, 8). A total of 473 non-duplicate systematic reviews (search #1) and 12,101 non-duplicate observational studies (search #2) were obtained from the search strategies. Full texts of 161 studies were assessed, and 54 prevalence studies were included in the meta-analysis (22–75). The PRISMA flowchart is presented in Figure 1.

Studies were published from 2001 to 2020, with most papers published after 2014 (*n* = 39). All studies had a cross-sectional design, and all but one study was conducted in the community (one study was conducted online). Sixteen studies were conducted in Iran, five in Pakistan, five in Egypt, five in Lebanon, three in Sudan, three in Saudi Arabia, three in Morocco, three in Iraq, two in Afghanistan, two in Jordan, two in Qatar, one in Bahrain, one in Palestine and one in the United Arab Emirates (UAE), while two studies reported prevalence estimates across multiple countries—Pakistan, Tunisia and UAE. No studies from Djibouti, Kuwait, Libya, Oman, Somalia, Syria, and Yemen were identified. The majority of studies used various screening tools to measure the prevalence of mental disorders, and 28 studies utilized diagnostic interviews. Further study characteristics are summarized in Appendix 6.

The sample size ranged from 450 (56) to 130,570 participants (64). The mean age range of participants was 21.8–73 years. Forty-seven studies focused on mental disorders in the general population, while seven were on trauma-exposed populations (*n* = 2 on earthquake survivors, *n* = 5 on refugees). The study sample sizes and main participant characteristics are further summarized in Appendix 7.

**Figure 2:**
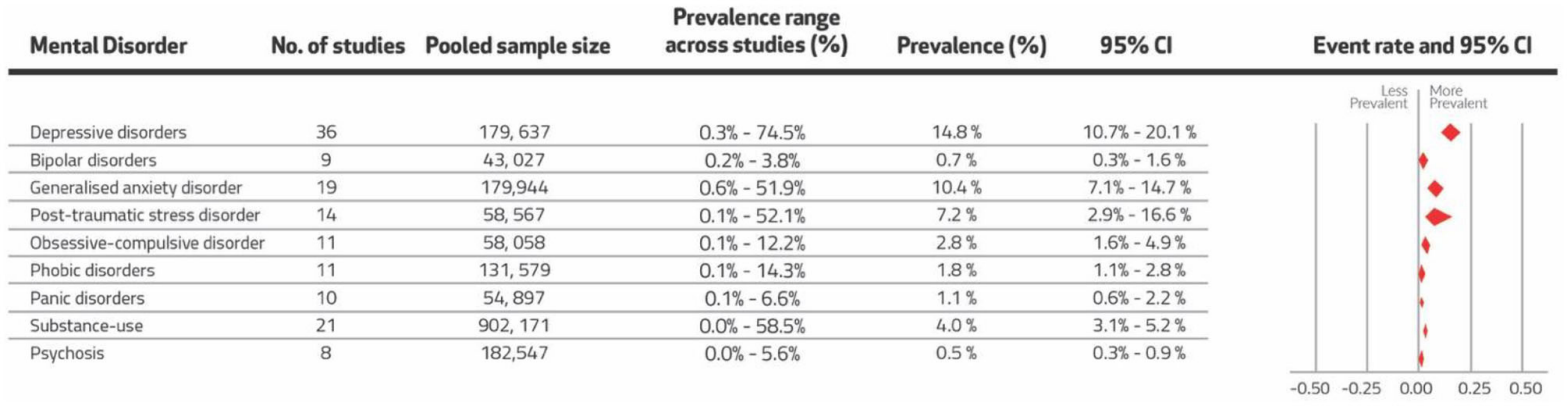
Random-effects pooled point prevalence estimates for mental disorders.

A total of 36 (67%; 41 study cohorts) reported on the prevalence of depression (*n* = 179,637), 9 (17%; 12 study cohorts) on bipolar disorder (*n* = 43,027), 19 (35%; 27 study cohorts) on GAD (*n* = 179,944), 14 (26%; 15 study cohorts) on PTSD (*n* = 58,567), 11 (20%) on OCD (*n* = 58,058), 11 (20%; 25 study cohorts) on phobic disorders (*n* = 131,579), 10 (19%) on panic disorders (*n* = 54,897), 21 (39%; 57 study cohorts) on substance use (*n* = 902,171) and 8 (15%; 18 study cohorts) on psychosis (*n* = 182,547). Figure 2 presents the pooled prevalence estimates (current, period or lifetime) for all mental disorders. Estimates per type of prevalence for each disorder are provided in Table 2.

**Table 2:** Mental disorders in the EMR by type of prevalence.

**Prevalence type**	**No. of** **cohorts**	**Prevalence** **(%)**	**95% CI**	***I***^**2**^ **(%)**
**Depressive disorder**				
Current	32	20.5	14.9–27.4%	99.78
Lifetime	6	4.2	1.8–9.6%	99.78
Period	3	4.3	1.2–13.5%	93.99
**Bipolar disorder**				
Current	6	1.9	0.1–3.0%	85.98
Lifetime	5	0.2	0.1–0.4%	19.63
Period	1	0.6	0.2–2.2%	0
**Generalized anxiety disorder**				
Current	12	10.3	6.1–17.0%	99.89
Lifetime	10	4.5	2.4–8.3%	99.42
Period	5	6.0	2.5–13.7%	98.04
**Post-traumatic stress disorder**				
Current	11	9.5	4.2–20.1%	99.60
Lifetime	4	3.3	0.8–12.9%	99.50
**Post-traumatic stress disorder**				
Current	5	2.7	1.1–6.6%	99.13
Lifetime	4	2.2	0.8–6.3%	99.20
Period	1	1.4	0.2–10.0%	0
**Phobic disorders**				
Current	11	2.6	1.3–5.2%	99.33
Lifetime	12	1.2	0.6–2.5%	99.17
Period	2	1.7	0.3–10.4%	94.37
**Panic disorders**				
Current	3	0.9	0.0–0.3%	90.95
Lifetime	6	1.3	0.1–0.3%	98.97
Period	1	0.9	0.0–0.7%	0
**Substance use**				
Current	10	8.4	4.9–14.1%	99.94
Lifetime	30	3.3	2.4–4.7%	99.95
Period	17	1.3	0.8–2.1%	99.64
**Psychosis**				
Current	5	1.3	0.4–4.1%	97.51
Lifetime	8	0.3	0.1–0.7%	98.87
Period	5	0.4	0.1–1.3%	24.69

### Depression

The prevalence for current, lifetime and period prevalence were 20.5% (95% CI: 14.9–27.4%), 4.2% (95% CI: 1.8–9.6%), and 4.3% (95% CI: 1.2–13.5%), respectively (Table 2). The random-effects pooled prevalence of depressive disorders was estimated at 14.8% (95% CI: 10.7–20.1%) (Figure 2). Substantial heterogeneity was present (*I*^2^ = 99.81%, τ^2^ = 1.44). The meta-analysis is presented in Appendix 8 (Forest plot Appendix 8.1). The sensitivity analyses did not reveal any significant changes in the prevalence of depressive disorders and the Egger's test revealed no evidence of publication bias (*p* = 0.84). The funnel plot is given in Appendix 8.2. The results from subgroup analyses and meta-regression are presented in Appendices 8.3, 8.4, respectively. Subgroup analyses revealed major differences between the prevalence reported in studies using diagnostic interviews compared to those using screening tools (*p* < 0.01), with studies using screening tools reporting four times the prevalence of depression (32.0%, 95% CI: 21.0–45.0%) than studies which employed diagnostic interviews (8.0%, 95% CI: 5.0–12.0%). Of the EMR countries, Afghanistan (33.0%, 95% CI: 7.0–75.0%), Egypt (22.0%, 95% CI: 8.0–47.0%), Jordan (37.0%, 95% CI: 9.0–78.0%), Lebanon (17.0%, 95% CI: 4.0–47.0%), Morocco (27.0%, 95% CI: 3.0–82.0%), Qatar (50.0%, 95% CI: 7.0–93.0%), and Sudan (40.0%, 95% CI: 10.0–80.0%) reported the highest prevalence, but difference in prevalence between the countries did not attain significance (*p* = 0.46). Additionally, higher prevalence of depression was reported among populations which were exposed to adversity, (33.0%, 95% CI: 11.0–66.0%), living under an authoritarian regime (16.0%, 95% CI: 11.0–23.0%), and living in low-income countries (28.0%, 95% CI: 11.0–54.0%). However, there was no evidence of a significant difference in prevalence estimates within these respective subgroups (*p* = 0.11, 0.74, 0.44, respectively). The meta-regression revealed no evidence of an association of depressive disorders with mean age (*p* = 0.34) or with country GDP (*p* = 0.10).

### Bipolar Disorders

The prevalence for current, lifetime and period prevalence were 1.9% (95% CI: 0.1–3.0%), 0.2% (95% CI: 0.1–0.4%), and 0.6% (95% CI: 0.2–2.2%), respectively (Table 2). The random-effects pooled prevalence of bipolar disorders was estimated at 0.7% (95% CI: 0.3–1.6%) (Figure 2). Substantial heterogeneity was evident in the reporting of this outcome (*I*^2^ = 97.82%). Results for bipolar disorders are presented in Appendix 9 (Forest plot in Appendix 9.1). The sensitivity analyses did not reveal any significant changes in the prevalence of bipolar disorders. However, the Egger's test showed evidence of publication bias (*p* = 0.08) (funnel plot in Appendix 9.2). Results from subgroup analyses and meta-regression are presented in Appendices 9.3, 9.4, respectively. Subgroup analyses did not reveal any significant differences between EMR countries (*p* = 0.89), regime types (*p* = 0.58) or country's income group (*p* = 0.80). Additionally, the meta-regression showed no evidence of an association of bipolar disorder with mean age (*p* = 0.14) or with country GDP (*p* = 0.38).

### Generalized Anxiety Disorder

The prevalence for current, lifetime and period prevalence were 10.3% (95% CI: 6.1–17.0%), 4.5% (95% CI: 2.4–8.3%), and 6.0% (95% CI: 2.5–13.7%), respectively (Table 2). The random-effects pooled prevalence of GAD was estimated at 6.9% (95% CI: 4.5–10.4%) (Figure 2). Substantial heterogeneity was present (*I*^2^ = 99.86%, τ^2^ = 1.37). Results for GAD are included in Appendix 10 (Forest plot presented in Appendix 10.1). The sensitivity analysis did not reveal any significant changes in the prevalence of GAD. However, the Egger's test revealed evidence of publication bias (*p* < 0.01) (funnel plot presented in Appendix 10.2). After adjusting for publication bias, the GAD's pooled prevalence was estimated as 10.4% (95% CI: 7.1–14.7%). The results from subgroup analyses and meta-regression are presented in Appendices 10.3, 10.4, respectively. Subgroup analyses revealed that Afghanistan (25.0%, 95% CI: 6.0–62.0%), Saudi Arabia (19.0%, 95% CI: 2.0–69.0%), Palestine (14.0%, 95% CI: 2.0–60.0%) and Iran (11.0%, 95% CI: 5.0–22.0%) had the highest prevalence of GAD. However, there was no evidence of a significant difference in prevalence between countries (*p* = 0.18). Additionally, there was no evidence of differences between regime types (*p* = 0.69). Low-income countries reported the highest GAD prevalence, but there was no evidence of a statistically significant difference between the countries based on their income levels (*p* = 0.11). There was some evidence of a difference between screening methods (*p* = 0.07), with studies using screening tools reporting double the prevalence (13.0%, 95% CI: 6.0–26.0%) than diagnostic interviews (6.0%, 95% CI: 4.0–9.0%). Studies including trauma-exposed populations reported double the prevalence of GAD (14.0%, 95% CI: 2.0–61.0%) than the general population (7.0%, 95% CI: 4.0–10.0%), but there was no evidence of a significant difference between the two population groups (*p* = 0.52). Additionally, the meta-regression revealed no evidence of an association of GAD with mean age (*p* = 0.13) or with country GDP (*p* = 0.98).

### Post-traumatic Stress Disorder

The prevalence for current and lifetime prevalence were 9.5% (95% CI: 4.2–20.1%) and 3.3% (95% CI: 0.8–12.9%) respectively (Table 2). The random-effects pooled prevalence of PTSD was estimated at 7.2% (95% CI: 2.9–16.6%) (Figure 2). Substantial heterogeneity was present (*I*^2^ = 99.73%, τ^2^ = 3.43). Results for PTSD are presented in Appendix 11 (forest plot included in Appendix 11.1). Sensitivity analysis did not reveal any significant changes in the prevalence of PTSD, and the Egger's test revealed no evidence of publication bias (*p* = 0.16) (funnel plot presented in Appendix 11.2). Results from subgroup analyses and meta-regression are presented in Appendices 11.3, 11.4, respectively. Subgroup analyses showed that Afghanistan (35.0%, 95% CI: 4.0–87.0%), Palestine (18.0%, 95% CI: 1.0–88.0%), and Sudan (36.0%, 95% CI: 2.0–95.0%) had the highest prevalence of PTSD. However, there was no evidence of a difference in prevalence between EMR countries (*p* = 0.15). Studies that used screening tools reported over double the prevalence of PTSD (17.0%, 95% CI: 3.0–61.0%) compared to studies that employed diagnostic interviews (6.0%, 95% CI: 2.0–15.0%), but there was no evidence of a significant difference between the two (*p* = 0.33). Generally, there was evidence of higher prevalence of PTSD in low-income countries (*p* = 0.03) and some evidence that trauma-exposed populations had over six times the prevalence of PTSD (33.0%, 95% CI: 6.0–80.0%) than the general population (5.0%, 95% CI: 2.0–13.0%) (*p* = 0.05). The meta-regression revealed no evidence of an association of PTSD with mean age (*p* = 0.88). However, there was evidence of a negative association of PTSD with country GDP (*p* = 0.04).

### Obsessive Compulsive Disorder

The prevalence for current, lifetime and period prevalence were 2.7% (95% CI: 1.1–6.6%), 2.2% (95% CI: 0.8–6.3%), and 1.4% (95% CI: 0.2–10.0%), respectively (Table 2). The random-effects pooled prevalence of OCD was estimated at 2.8% (95% CI: 1.6–4.9%) (Figure 2). Substantial heterogeneity was evident in the reporting of this outcome (*I*^2^ = 99.01%, τ^2^ = 0.85). Results for OCD are presented in Appendix 12 (forest plot included in Appendix 12.1). Sensitivity analysis did not reveal any significant changes in the prevalence of OCD, and the Egger's test revealed no evidence of publication bias (*p* = 0.87) (funnel plot in Appendix 12.2). Results from the subgroup analyses and meta-regression are presented in Appendices 12.3, 12.4, respectively. Subgroup analyses revealed evidence of a difference between EMR countries (*p* = 0.01) with Morocco (6.0%, 95% CI: 2.0–21.0%), Iran (6.0%, 95% CI: 2.0–14.0%), and Iraq (5.0%, 95% CI: 1.0–25.0%) reporting higher prevalence of OCD. There was also evidence of prevalence differing across countries based on their income groups (*p* = 0.01), with high-income countries reporting the lowest prevalence of OCD (0.1%, 95% CI: 0.0–1.0%). The meta-regression revealed no evidence of an association of OCD with mean age (*p* = 0.66) or with country GDP (*p* = 0.74).

### Phobic Disorders

The prevalence for current, lifetime and period prevalence were 2.6% (95% CI: 1.3–5.2%), 1.2% (95% CI: 0.6–2.5%), and 1.7% (95% CI: 0.3–10.4%), respectively (Table 2). The random-effects pooled prevalence of phobic disorders was estimated at 1.8% (95% CI: 1.1–2.8%) (Figure 2). Substantial heterogeneity was evident in the reporting of this outcome (*I*^2^ = 99.23%, τ^2^ = 1.35). Results for phobic disorders are included in Appendix 13 (forest plot presented in Appendix 13.1). Sensitivity analysis did not reveal any significant changes in the prevalence of phobic disorders, and the Egger's test revealed no evidence of publication bias (*p* = 0.13) (funnel plot included in Appendix 13.2). Results from the subgroup analyses and meta-regression are presented in Appendices 13.3, 13.4, respectively. Subgroup analyses revealed strong evidence for differences in the prevalence of phobic disorders across EMR countries (*p* < 0.01), with the highest prevalence in Sudan (14.0%, 95% CI: 4.0–39.0%) and Morocco (8.0%, 95% CI: 4.0–13.0%). There was also evidence of differences across country income groups (*p* < 0.01), with the highest prevalence reported in low-income countries (14.0%, 95% CI: 26.0–58.0%). Additionally, there was evidence of a difference across population type (*p* = 0.04) and regime type (*p* = 0.01), with populations exposed to adversity having seven times the prevalence of phobic disorders (14.0%, 95% CI: 2.0–60.0%) than the general population (2.0%, 95% CI: 1.0–3.0%), and populations living under hybrid regimes having four times the prevalence (4.0%, 95% CI: 2.0–8.0%) than those under authoritarian regimes (1.0%, 95% CI: 1.0–2.0%). The meta-regression revealed no evidence of an association of phobic disorders with mean age (*p* = 0.15), but there was evidence of a negative association with country GDP (*p* = 0.04).

### Panic Disorders

The prevalence for current, lifetime and period prevalence were 0.9% (95% CI: 0.0–0.3%), 1.3% (95% CI: 0.1–0.3%), and 0.9% (95% CI: 0.0–0.7%), respectively (Table 2). The random-effects pooled prevalence of panic disorders was estimated at 1.1% (95% CI: 0.6–2.2%) (Figure 2). Substantial heterogeneity was evident in the reporting of this outcome (*I*^2^ = 98.69%, τ^2^ = 1.09). Results for panic disorders are presented in Appendix 14 (forest plot included in Appendix 14.1). Sensitivity analysis did not reveal any significant changes in the prevalence of panic disorders, and the Egger's test showed no evidence of publication bias (*p* = 0.31) (funnel plot in Appendix 14.2). Results from the subgroup analyses and meta-regression are presented in Appendices 14.3, 14.4, respectively. Subgroup analyses revealed evidence for differences in the prevalence of panic disorders across EMR countries (*p* < 0.01), with Morocco reporting the highest prevalence (4.0%, 95% CI: 2.0–10.0%). There was also evidence of a difference between regime type (*p* = 0.02), with populations living under hybrid regimes reporting a higher prevalence of panic disorders (3.0%, 95% CI: 1.0–5.0%) than those under authoritarian regimes (1.0%, 95% CI: 1.0–2.0%). The meta-regression revealed no evidence of an association of panic disorders with country GDP (*p* = 0.16).

### Substance Use

The prevalence for current, lifetime and period prevalence were 8.4% (95% CI: 4.9–14.1%), 3.3% (95% CI: 2.4–4.7%), and 1.3% (95% CI: 0.8–2.1%), respectively (Table 2). The random-effects pooled prevalence of substance use was estimated at 3.0% (95% CI: 2.3–3.9%) (Figure 2). Substantial heterogeneity was evident in the reporting of this outcome (*I*^2^ = 99.94%, τ^2^= 1.02). Results for substance use are presented in Appendix 15 (forest plot included in Appendix 15.1). Sensitivity analysis did not reveal any significant changes in the prevalence of substance use. Among eight of these studies, pooled prevalence for tobacco use alone was estimated at 23.3% (95% CI: 19.2–28.1%, *I*^2^ = 99.85%, 280,826) (Appendix 15.5).

The Egger's test revealed strong evidence of publication bias (*p* < 0.01) (funnel plot is presented in Appendix 15.2). After adjusting for publication bias, the pooled prevalence of substance use was estimated at 4.02% (95% CI: 3.1–5.2%). Results from the subgroup analyses and meta-regression are presented in Appendices 15.3, 15.4, respectively. Subgroup analyses revealed strong evidence for differences in the prevalence of substance use across EMR countries (*p* < 0.01), with Pakistan (32.0%, 95% CI: 6.0–78.0%), Qatar (29.0%, 95% CI: 5.0–75.0%), Morocco (15.0%, 95% CI: 8.0–26.0%), and Saudi Arabia (14.0%, 95% CI: 5.0–34.0%) reporting the highest prevalence. There was also strong evidence of differences across country income subgroups (*p* < 0.01), with high-income countries reporting the highest prevalence (10.0%, 95% CI: 4.0–21.0%). The prevalence also differed across regime types and screening method (*p* < 0.01 for both), with hybrid regimes and studies using screening tools as opposed to diagnostic interviews reporting higher prevalence of substance use (13.0%, 95% CI: 7.0–22.0% and 8.0%, 95% CI: 6.0–11.0%, respectively). The meta-regression also revealed evidence of a positive association of substance use with mean age (*p* < 0.01). However, country GDP was not found to have a significant association with substance use (*p* = 0.55).

### Psychosis

The prevalence for current, lifetime and period prevalence were 1.3% (95% CI: 0.4–4.1%), 0.3% (95% CI: 0.1–0.7%), and 0.4% (95% CI: 0.1–1.3%), respectively (Table 2). The random-effects pooled prevalence of psychosis was estimated at 0.5% (95% CI: 0.3–0.9%) (Figure 2). Substantial heterogeneity was evident in the reporting of this outcome (*I*^2^ = 97.93%). Results for psychosis are included in Appendix 16 (forest plot included in Appendix 16.1). Sensitivity analysis did not reveal any significant changes in the prevalence of psychosis, and the Egger's test revealed no evidence of publication bias (*p* = 0.42) (funnel plot is included in Appendix 16.2). Results from the subgroup analyses and meta-regression are presented in Appendices 16.3, 16.4, respectively. Subgroup analyses revealed a higher prevalence of psychosis in low-income countries (5.6%, 95% CI: 0.8–30.8%). However, there was no evidence of a difference between the prevalence of psychosis across country's income group or EMR countries and screening method (*p* > 0.05). The meta-regression revealed no evidence of an association of psychosis with mean age (*p* = 0.38) or with country GDP (*p* = 0.32).

### Quality Assessment

The quality assessment of all included studies is presented in Appendix 17. Most studies were rated as medium-quality (*n* = 28), with 22 rated as high-quality and only four rated as low-quality. Across the items, all studies had an adequate sample size (*n* = 54). Additionally, the majority of studies had an appropriate sampling frame (*n* = 50) and sampling method (*n* = 49), appropriate statistics (*n* = 48) and appropriate details of the sample and setting (*n* = 42). Fewer studies conducted data analysis with sufficient coverage (*n* = 29), employed valid (*n* = 34) and reliable (*n* = 33) measures, and had adequate response rates (*n* = 27), that is, ≥ 85%.

## Discussion

The current review included 54 studies that reported prevalence estimates for mental and substance use disorders across 15 countries in the WHO EMR. Depressive disorders showed the highest pooled prevalence of current, period and lifetime prevalence (14.8%, 95% CI: 10.7–20.1%), followed by GAD (10.4%, 95% CI: 7.1–14.7%), PTSD (7.2%, 95% CI: 2.9–16.6%), substance use (4.0%, 95% CI: 3.1–5.2%), OCD (2.8%, 95% CI: 1.6–4.9%), phobic disorders (1.8%, 95% CI: 1.1–2.8%), panic disorders (1.1%, 95% CI: 0.6–2.2%), bipolar disorders (0.7%, 95% CI: 0.3–1.6%), and psychosis (0.5%, 95% CI: 0.3–0.9%). Period and lifetime prevalence showed little difference across mental disorders. More pronounced differences in prevalence were seen for depression and GAD, specifically between current and lifetime prevalence depression: current prevalence 20.5% (95% CI: 14.9–27.4%), vs. lifetime prevalence: 4.2% (95%CI: 1.8–9.6%); GAD: current prevalence 10.3% (95% CI: 6.1–17.0), vs. lifetime prevalence: 4.5% (95% CI: 2.4–8.3%).

Our findings correspond to the current evidence base. For example, depression was the most prevalent disorder within our review, a finding that is consistent with previous systematic reviews in the EMR (76) and the regional prevalence recently reported by WHO (77). The prevalence of depression in the EMR is higher than the global prevalence (4.7%, 95% CI: 4.4–5.0%) (15), which can be explained by the ongoing conflict and unrest in the majority of the EMR countries (78, 79). Similarly, our pooled prevalence estimate for bipolar disorder (0.7%, 95% CI: 0.3–1.6%) is consistent with the global prevalence estimate of 0.74% (14). Other reviews have found that the prevalence of bipolar disorders is between 1 and 2% (13, 80). Our period prevalence for GAD is 6.0% (95% CI: 2.5–13.7%) which corresponds with the period prevalence for anxiety disorders (including GAD) reported by Steel et al. (6.7%, 95% CI: 6.1–7.9%) (2). Similarly, the lifetime prevalence for PTSD which we have found (3.3%, 95% CI: 0.8–12.9%) corresponds to the WMHS estimate of 3.2% (95% CI: 3.0–3.4%) (81). For OCD, the period prevalence (1.4%, 95% CI: 0.2–10.0%) and lifetime prevalence (2.2%, 95% CI: 0.8–6.3%) obtained in this review are greater than previous global 1-year 0.54% (95% CI: 0.28–0.86%) and lifetime estimate (1.3%; 95% CI: 0.86–1.8%) as reported by Somers et al. (12). As for phobic disorders, the period prevalence (1.7%, 95% CI: 0.3–10.4%) and lifetime prevalence (1.2%, 95% CI: 0.6–2.5%) obtained in our review is also less than Somers's et al. 1-year (3.0%, 95% CI: 0.98–5.8%) and lifetime estimates (5.3%, 95% CI: 3.4–7.9%) (12). However, our estimates for panic disorders, specifically the period prevalence estimate (0.9%, 95% CI: 0.0–0.7%) corresponds to findings by Somers et al. (0.99%, 95% CI: 0.55–1.5%). Furthermore, our lifetime estimate for panic disorders (1.3%, 95% CI: 0.1–0.3%) is similar to Somer et al.'s lifetime estimates (1.2%, 95% CI: 0.7–1.9%) (12). Although there seems to be consistency in prevalence estimates for panic disorders across regions, overall, there is a paucity of evidence on OCD, phobic and panic disorders and therefore, any variations as well as similarities in the results require further investigation into the source of these trends. The pooled prevalence of substance use after adjusting for publication bias (4.0%, 95% CI: 3.1–5.2%) is similar to global period prevalence estimates (3.8%, 95% CI: 3.3–4.2%) (2). Additionally, the global lifetime prevalence estimate obtained by other authors (10.7%, 95% CI: 9.2–12.5%) (2) is higher than the lifetime prevalence estimate obtained in the current study (3.3%, 95% CI: 2.4–4.7%). Research on substance use faces challenges within the region due to stigma and their illegal status and therefore, it may be difficult to assess their true prevalence. Psychosis shows the lowest pooled prevalence among all mental disorders (0.5%, 95% CI: 0.3–0.9%), confirming global trends. Psychosis has been reported higher in conflict-affected settings with an established association between psychotic symptoms and PTSD (82).

### Subgroup Analyses and Meta-Regression

Subgroup analyses on EMR countries, population type, regime type, screening method (diagnostic or non-diagnostic), and country's income group revealed possible reasons for the heterogeneity observed. The prevalence of OCD, panic disorders, phobic disorders and substance use varied significantly across EMR countries. Additionally, populations exposed to adversity had a higher prevalence (between two to seven times) than the general population. However, this difference was only significant for phobic disorders and marginally significant for PTSD. Higher rates of depressive disorders, GAD, PTSD, phobic disorders and psychosis were observed in low-income countries, with evidence of a difference in the prevalence of PTSD and phobic disorders across income groups.

Additionally, individuals in hybrid regimes were found to be significantly more likely to show phobic and panic disorders, as well as substance use. Substance use was also found to be significantly higher in high-income countries. Finally, studies that used screening tools reported a consistently higher prevalence of mental disorders than studies that used diagnostic interviews. Significantly higher rates were reported for depressive disorders and substance use and marginally significantly higher rates for GAD.

In addition to subgroup analyses, meta-regression analyses were also performed to assess the variation in prevalence estimates of mental disorders and substance use based on the age of participants and country GDP. Meta-regression analyses in the current review revealed that substance use was positively associated with mean age. Furthermore, country GDP was found to be negatively associated with both PTSD and phobic disorders. A discussion on further trends across other country-level factors, age of participants and population type is provided in Appendix 18.

### Strengths and Limitations

This study synthesizes current evidence on the prevalence of mental and substance use disorders within the EMR, with a search strategy adapted from previous reviews (8, 83). However, title and abstract screening was predominately done by one author, with another author double screening 40% of the articles only. This selection process was chosen due to time constraints and may have increased the chances of missing relevant studies. On the other hand, all full texts were reviewed by at least two authors working independently from each other. We did not search for gray literature and conducted the literature search in English only. Furthermore, we did not search for cross-sectional studies before 2014 and relied on data from systematic reviews published until then. Therefore, it may be possible that some individual studies were missed.

At the data extraction stage, lifetime prevalence estimates were preferred over current or period prevalence rates (rational provided in Annex 5). However, lifetime prevalence estimates may be more susceptible to recall bias due to a more extended period of recall (2). Furthermore, we combined current, period and lifetime prevalence estimates (Figure 2) to be able to include a larger number of studies per disorder type but separate analyses were also conducted taking account of possible differences in prevalence (see Table 2). However, some of these are based on a low number of studies which may hamper generalisability of findings. Prevalence estimates for some of the mental disorders were very low, and even larger sample sizes (>500) had higher margin of errors and imprecise estimates, rendering some of our pooled estimates (Figure 2) asymmetric. Additionally, substance use included tobacco use, which generally has a high population prevalence and may have affected the overall prevalence estimate (2) (forest plot for tobacco use included in the Supplementary Material). Separate analyses by type of prevalence was also being conducted. Period and lifetime prevalence showed little difference across mental disorders. More pronounced differences in prevalence were seen for depression and GAD and these differences between current and lifetime prevalence for depression and GAD may be due clinical and statistical heterogeneity across different studies. This disparity in finding is seen due to a broader set of inclusion and exclusion criteria, leading to heterogeneity in characteristics of study samples, screening and diagnostic methods and thresholds considered for screening scales. For future studies, we recommend that investigators consider narrower inclusion and exclusion criteria pertaining to more homogenous study populations and research methods, to arrive at more robust prevalence estimates.

There are a few methodological factors that need to be considered as well. First, around 28% of the studies had an under-representation of adults aged 50+ years which may affect the generalisability of the findings to older age-groups. Second, around 13% of studies were rated to have an inadequate response rate (< 85%), and 37% of the studies did not specify the response rate. This may have led to more conservative estimates as non-respondents may have potentially higher rates of mental disorders (84, 85). Third, substantial heterogeneity was identified in all random effect models, with *I*^2^ exceeding 95% in each of them. Heterogeneity can have an adverse effect on the inter-survey comparability and the reliability and interpretability of the pooled prevalence estimates. However, sensitivity analyses yielded no significant changes in any of the estimates, thereby confirming the stability of the prevalence estimates to a large extent. Finally, the majority of studies employed screening tools only which may have overestimated prevalence of mental disorders.

## Conclusion

The prevalence of mental and substance use disorders in the EMR is high. Despite substantial inter-survey heterogeneity, our estimates align with previous data on mental and substance use disorders at the global and regional level. Our systematic review and meta-analysis provide important new evidence to better understand the burden of mental and substance use disorders in the EMR, which may facilitate future mental health research and policymaking in this region.

## Data Availability Statement

The original contributions presented in the study are included in the article/Supplementary Material, further inquiries can be directed to the corresponding author/s.

## Author Contributions

AZ conducted the literature search, extracted and analyses data, and drafted the paper. AW, SN, and MH assisted in data extraction, analyzed, and interpretation of data. AR, KS, and DF critically revised the paper. All authors reviewed the paper and approved the final version.

## Conflict of Interest

The authors declare that the research was conducted in the absence of any commercial or financial relationships that could be construed as a potential conflict of interest.
