# Supplementary material for: Prevalence of Mental Disorders in the WHO Eastern Mediterranean Region: A Systematic Review and Meta-Analysis
Source: Front Psychiatry. 2021 Jul 14;12:665019. doi: 10.3389/fpsyt.2021.665019 (PMC8316754; doi:10.3389/fpsyt.2021.665019)
Supplement: Supplementary file 1 [file Data_Sheet_1.pdf]

Appendices

Annex 1: List of countries in the EMR region of WHO

Afghanistan, the Arab Republic of Egypt (Egypt), Bahrain, Djibouti, Iraq, the Islamic Republic of Iran (Iran), Jordan, the Kingdom of Saudi Arabia (Saudi Arabia), Kuwait, Lebanon, Libya, Morocco, Oman, Pakistan, Palestine, Qatar, the Republic of Yemen (Yemen), Somalia, Sudan, the Syrian Arab Republic (Syria), Tunisia, and the United Arab Emirates (UAE)

Annex 2: PRISMA checklist

Section/topic	#	Checklist item	Reported on page #
**TITLE**			
Title	1	Identify the report as a systematic review, meta-analysis, or both.	1
**ABSTRACT**			
Structured summary	2	Provide a structured summary including, as applicable: background; objectives; data sources; study eligibility criteria, participants, and interventions; study appraisal and synthesis methods; results; limitations; conclusions and implications of key findings; systematic review registration number.	2
**INTRODUCTION**			
Rationale	3	Describe the rationale for the review in the context of what is already known.	3
Objectives	4	Provide an explicit statement of questions being addressed with reference to participants, interventions, comparisons, outcomes, and study design (PICOS).	3
**METHODS**			
Protocol and registration	5	Indicate if a review protocol exists, if and where it can be accessed (e.g., Web address), and, if available, provide registration information including registration number.	4
Eligibility criteria	6	Specify study characteristics (e.g., PICOS, length of follow-up) and report characteristics (e.g., years considered, language, publication status) used as criteria for eligibility, giving rationale.	4, Annex 3
Information sources	7	Describe all information sources (e.g., databases with dates of coverage, contact with study authors to identify additional studies) in the search and date last searched.	4
Search	8	Present full electronic search strategy for at least one database, including any limits used, such that it could be repeated.	Annex 4
Study selection	9	State the process for selecting studies (i.e., screening, eligibility, included in systematic review, and, if applicable, included in the meta-analysis).	5-6
Data collection process	10	Describe method of data extraction from reports (e.g., piloted forms, independently, in duplicate) and any processes for obtaining and confirming data from investigators.	5-6

Data items	11	List and define all variables for which data were sought (e.g., PICOS, funding sources) and any assumptions and simplifications made.	7, Append. 7
Risk of bias in individual studies	12	Describe methods used for assessing risk of bias of individual studies (including specification of whether this was done at the study or outcome level), and how this information is to be used in any data synthesis.	6
Summary measures	13	State the principal summary measures (e.g., risk ratio, difference in means).	5
Synthesis of results	14	Describe the methods of handling data and combining results of studies, if done, including measures of consistency (e.g., I^2) for each meta-analysis.	5-6

Annex 3: Inclusion and exclusion criteria

	Inclusion criteria	Exclusion criteria
Mental disorder	Depressive disorders, bipolar disorders, generalised anxiety disorder, post-traumatic stress disorder, psychosis (psychotic disorders or psychotic experiences), substance use	Comorbidity with other physical health problems or chronic illnesses
Population	Adult population, that is, individuals aged 18 years or older	Studies were excluded if they primarily focused on children or adolescent populations, as well as racial, ethnic, occupational or military subgroups only
Setting	WHO-defined Eastern Mediterranean Region (see Annex 1 for list of countries)<br><br>Studies conducted in community settings, the general population, or refugee settings (e.g. camps)	All other countries not in EMROStudies conducted in non-community settings such as hospitals
Study design	Cross-sectional and prospective cohort studies	All other study designs, such as, but not limited to qualitative and case-control studies, randomised controlled trials
Outcomes	Current (1- or 2- week, or 1 month), Period (6 or 12-months) or lifetime prevalence of the above-mentioned mental disorders assessed by either diagnostic interviews or validated screening tools.	Studies were excluded if prevalence of the above-mentioned mental disorders were not reported
Papers	Only studies published in peer-reviewed journal articles which were written in English were included.	Editorials, commentaries, case reports, clinical guidelines, book chapters, conference abstracts or posters, and dissertations were excluded.<br><br>Grey literature was excluded.<br><br>Papers in any language other than English were excluded.

Annex 4: Search strategy (as used in PubMed)

	Search Query
1	((((("Prevalence" OR "Frequency" OR "epidemiology" OR "epidemiological" OR "proportion" OR "cases" OR "Odds" OR " Risks" OR "Status" OR "Associated factor" OR "Distribution" OR "Determinants" OR " Risk factor" OR correlate* OR predictor*)) AND (psych* OR "Mental" OR "mood disorders" OR "Depression" OR "Depressive disorder" OR "Substance abuse" OR "substance-use" OR "substance-related disorders" OR "posttraumatic stress disorder" OR "post-traumatic stress disorder" OR "PTSD" OR "obsessive compulsive disorder" OR "OCD" OR "bipolar disorder" OR "Anxiety" OR "Panic disorder" OR "schizophrenia" OR "GAD" OR "Acute stress disorder")) AND ("Global"[Title/Abstract] OR "EMR"[Title/Abstract] OR "EMRO"[Title/Abstract] OR "Eastern Mediterranean Region"[Title/Abstract] OR "Middle East"[Title/Abstract] OR "Middle Eastern Countries"[Title/Abstract] OR "Northern Africa"[Title/Abstract] OR "North Africa"[Title/Abstract] OR "South Asia"[Title/Abstract] OR "Western Asia"[Title/Abstract] OR "Afghanistan"[Title/Abstract] OR "Bahrain"[Title/Abstract] OR "Djibouti"[Title/Abstract] OR "Egypt"[Title/Abstract] OR "Iran"[Title/Abstract] OR "Iraq"[Title/Abstract] OR "Jordan"[Title/Abstract] OR "Kuwait"[Title/Abstract] OR "Lebanon"[Title/Abstract] OR "Libya"[Title/Abstract] OR "Morocco"[Title/Abstract] OR "Oman"[Title/Abstract] OR "Pakistan"[Title/Abstract] OR "Palestine"[Title/Abstract] OR "Qatar"[Title/Abstract] OR "Saudi Arabia"[Title/Abstract] OR "Somalia"[Title/Abstract] OR "Sudan"[Title/Abstract] OR "Syria"[Title/Abstract] OR "Tunisia"[Title/Abstract] OR "United Arab Emirates"[Title/Abstract] OR "UAE"[Title/Abstract] OR "Yemen"[Title/Abstract])) AND ("Systematic Review"[Title] OR "meta-analysis"[Title] OR "pooled estimate"[Title] OR "pooled effect"[Title] OR "Systematic Literature Review"[Title] OR "Meta-regression"[Title] OR "meta-analytic"[Title]))
2	((((("Prevalence" OR "Frequency" OR "epidemiology" OR "epidemiological" OR "proportion" OR "cases" OR "Odds" OR " Risks" OR "Status" OR "Associated factor" OR "Distribution" OR "Determinants" OR " Risk factor" OR correlate* OR predictor*)) AND (psych* OR "Mental" OR "mood disorders" OR "Depression" OR "Depressive disorder" OR "Substance abuse" OR "substance-use" OR "substance-related disorders" OR "posttraumatic stress disorder" OR "post-traumatic stress disorder" OR "PTSD" OR "obsessive compulsive disorder" OR "OCD" OR "bipolar disorder" OR "Anxiety" OR "Panic disorder" OR "schizophrenia" OR "GAD" OR "Acute stress disorder")) AND ("Global"[Title/Abstract] OR "EMR"[Title/Abstract] OR "EMRO"[Title/Abstract] OR "Eastern Mediterranean Region"[Title/Abstract] OR "Middle East"[Title/Abstract] OR "Middle Eastern Countries"[Title/Abstract] OR "Northern Africa"[Title/Abstract] OR "North Africa"[Title/Abstract] OR "South Asia"[Title/Abstract] OR "Western Asia"[Title/Abstract] OR "Afghanistan"[Title/Abstract] OR "Bahrain"[Title/Abstract] OR "Djibouti"[Title/Abstract] OR "Egypt"[Title/Abstract] OR "Iran"[Title/Abstract] OR "Iraq"[Title/Abstract] OR "Jordan"[Title/Abstract] OR "Kuwait"[Title/Abstract] OR "Lebanon"[Title/Abstract] OR "Libya"[Title/Abstract] OR "Morocco"[Title/Abstract] OR "Oman"[Title/Abstract] OR "Pakistan"[Title/Abstract] OR "Palestine"[Title/Abstract] OR "Qatar"[Title/Abstract] OR "Saudi Arabia"[Title/Abstract] OR "Somalia"[Title/Abstract] OR "Sudan"[Title/Abstract] OR "Syria"[Title/Abstract] OR "Tunisia"[Title/Abstract] OR "United Arab Emirates"[Title/Abstract] OR "UAE"[Title/Abstract] OR "Yemen"[Title/Abstract])) AND (cross-section*[Title/Abstract] OR "longitudinal"[Title/Abstract] OR "Prospective"[Title/Abstract] OR cohort*[Title/Abstract]))

Note: Search query 1 had the years limited till 2013 and search query 2 had the years limited to between 2014 and 2020.

Annex 5: Type of data extracted

Data was extracted on the following: first author's last name and year of publication, study setting, geographical scope, participant education and income level, population group, age range and mean-age of participants, country of study and it's World Bank income classification, GDP (gross domestic product) per capita (2019 estimates expressed in United States (US) dollars), country regime type, percentage of males in the population, mental disorder investigated, type of screening method and its mode of delivery, screening instruments and their cut-off values or diagnostic criteria, study sample size, the reported number of people with mental disorders and finally, the sampling method.

The aggregate number of participants with mental disorders was extracted. Additionally, if an article reported lifetime prevalence as well as period or current prevalence of mental disorder, the lifetime estimate was preferred. This is because majority of mental disorders have a relapsing and remitting course and period or current prevalence estimates may not capture individuals who are in remission for that period (but relapse at a different interval or time-point to the one in which the prevalence was calculated).

Appendix 6: Study characteristics of papers included in the meta-analysis

Author, Year	Geographical scope	Country	Country's income group	Country's GDP	Country regime type	Screening Method	Mode of screening method	Diagnostic Criteria	Sampling technique
El-Sherbiny et al., 2016	Mixed	Egypt	Lower-middle	303,175	Authoritarian	Non-diagnostic	Self-report	N/A	Random
Almuneef et al., 2016	Mixed	Saudi Arabia	High	792,967	Authoritarian	Diagnostic	Not specified	Medical	Convenience
Baniasadi et al., 2019	Urban	Iran	Upper-middle	445,345	Authoritarian	Non-diagnostic	Face-to-Face	N/A	Random
Gammouh et al., 2015	Urban	Jordan	Upper-middle	43,744	Authoritarian	Non-diagnostic	Self-report	N/A	Convenience
Aly et al., 2018	Mixed	Egypt	Lower-middle	303,175	Authoritarian	Non-diagnostic	Face-to-Face	N/A	Random
Tayefi et al., 2017	Urban	Iran	Upper-middle	445,345	Authoritarian	Non-diagnostic	Not specified	N/A	Random
Alvi et al., 2017	Mixed	Pakistan	Lower-middle	278,222	Hybrid	Non-diagnostic	Face-to-Face	N/A	Mixed
Azizi et al., 2019	Urban	Iran	Upper-middle	445,345	Authoritarian	Non-diagnostic	Face-to-Face	N/A	Random
Obeid et al., 2020	Rural	Lebanon	Upper-middle	53,367	Hybrid	Non-diagnostic	Face-to-Face	N/A	Random
Hamdan-Mansour et al., 2017	Mixed	Jordan	Upper-middle	43,744	Authoritarian	Non-diagnostic	Self-report	N/A	Random
Aoun et al., 2018	Not specified	Lebanon	Upper-middle	53,367	Hybrid	Non-diagnostic	Face-to-Face	N/A	Convenience
Al Rashed et al., 2019	Mixed	Saudi Arabia	High	792,967	Authoritarian	Diagnostic	Mixed	DSM-IV	Convenience
Farhood et al., 2016	Rural	Lebanon	Upper-middle	53,367	Hybrid	Non-diagnostic	Face-to-Face	N/A	Random
Alhassan et al., 2018	Mixed	Saudi Arabia	High	792,967	Authoritarian	Non-diagnostic	Web-based	N/A	Convenience
Rafiey et al., 2019	Rural	Iran	Upper-middle	445,345	Authoritarian	Diagnostic	Face-to-Face	DSM-V	Random
Farooq et al., 2019	Urban	Pakistan	Lower-middle	278,222	Hybrid	Non-diagnostic	Face-to-Face	N/A	Random
Salah et al., 2015	Mixed	Sudan	Low	18,902	Authoritarian	Diagnostic	Face-to-Face	MINI	Random
El-Gilany et al., 2018	Mixed	Egypt	Lower-middle	303,175	Authoritarian	Non-diagnostic	Face-to-Face	N/A	Random
Bakhtiari et al., 2018	Urban	Iran	Upper-middle	445,345	Authoritarian	Non-diagnostic	Not specified	N/A	Universal
Abou-Saleh et al., 2001	Urban	UAE	High	421,142	Authoritarian	Diagnostic	Face-to-Face	CIDI	Random
Mohammadi et al., 2005	Mixed	Iran	Upper-middle	445,345	Authoritarian	Diagnostic	Face-to-Face	DSM-IV	Random
Kim et al., 2007	Mixed	Sudan	Low	18,902	Authoritarian	Diagnostic	Face-to-Face	DSM-IV	Random
De Jong et al., 2003	Mixed	Palestine	Lower-middle	14,616	Authoritarian	Diagnostic	Face-to-Face	CIDI	Random
Bhamani et al., 2013	Urban	Pakistan	Lower-middle	278,222	Hybrid	Non-diagnostic	Face-to-Face	N/A	Random
Ghanem et al., 2009	Mixed	Egypt	Lower-middle	303,175	Authoritarian	Diagnostic	Face-to-Face	MINI	Random

Roberts et al., 2009	Urban	Sudan	Low	18,902	Authoritarian	Diagnostic	Face-to-Face	DSM-IV	Random
Kadri et al., 2007	Urban	Morocco	Lower-middle	118,725	Hybrid	Diagnostic	Face-to-Face	MINI	Random
Nisar et al., 2004	Not specified	Pakistan	Lower-middle	278,222	Hybrid	Diagnostic	Face-to-Face	MINI	Universal
Mufti et al., 2005	Rural	Afghanistan	Low	19,101	Authoritarian	Diagnostic	Face-to-Face	MINI	Universal
El-Wasify et al., 2011	Mixed	Egypt	Lower-middle	303,175	Authoritarian	Diagnostic	Face-to-Face	SCAN-PSE	Random
Alhasnawi et al., 2009	Mixed	Iraq	Upper-middle	234,094	Authoritarian	Diagnostic	Face-to-Face	CIDI	Random
Ahmadvand et al., 2012	Urban	Iran	Upper-middle	445,345	Authoritarian	Diagnostic	Face-to-Face	DSM-IV	Random
Kadri et al., 2010	Mixed	Morocco	Lower- middle	118,725	Hybrid	Diagnostic	Face-to-Face	MINI	Random
Scholte et al., 2004	Mixed	Afghanistan	Low	19,101	Authoritarian	Diagnostic	Face-to-Face	DSM-IV	Random
Karam et al., 2016	Mixed	Lebanon	Upper-middle	53,367	Hybrid	Diagnostic	Face-to-Face	CIDI	Proportionate
Rezaei et al., 2017	Urban	Iran	Upper-middle	445,345	Authoritarian	Non-diagnostic	Self-report	N/A	Convenience
Ruscio et al., 2017	Not specified	Iraq	Upper-middle	234,094	Authoritarian	Diagnostic	Face-to-Face	CIDI	Not specified
Mehrabi et al., 2019	Urban	Iran	Upper-middle	445,345	Authoritarian	Non-diagnostic	Self-report	N/A	Random
Khaled et al., 2020	Mixed	Qatar	High	183,466	Authoritarian	Diagnostic	Face-to-Face	CIDI	Random
Hasan et al., 2019	Urban	Pakistan	Lower-middle	278,222	Hybrid	Non-diagnostic	Face-to-Face	N/A	Convenience
Khazaie et al. 2019	Mixed	Iran	Upper-middle	445,345	Authoritarian	Diagnostic	Mixed	CIDI	Random
Degenhardt et al., 2019	Mixed	Lebanon	Upper-middle	53,367	Hybrid	Diagnostic	Face-to-Face	CIDI	Random
Khaled et al., 2019	Mixed	Qatar	High	183,466	Authoritarian	Non-diagnostic	Phone	N/A	Random
Kausar et al., 2015	Mixed	Pakistan	Lower-middle	278,222	Hybrid	Diagnostic	Face-to-Face	DSM-IV	Random
Noorbala et al., 2015	Mixed	Iran	Upper-middle	445,345	Authoritarian	Non-diagnostic	Face-to-Face	N/A	Random
Sharifi et al., 2017	Mixed	Iran	Upper-middle	445,345	Authoritarian	Non-diagnostic	Face-to-Face	N/A	Random
Stubbs et al., 2017	Mixed	Pakistan	Lowe-middle	278,222	Hybrid	Non-diagnostic	Face-to-Face	N/A	Random
	Mixed	Tunisia	Lower-middle	38,798	Flawed Democracy	Non-diagnostic	Face-to-Face	N/A	Random
	Mixed	UAE	High	421,142	Authoritarian	Non-diagnostic	Face-to-Face	N/A	Random
Rafiey et al., 2017	Urban	Iran	Upper-middle	445,345	Authoritarian	Diagnostic	Face-to-Face	DSM-IV	Not specified
Laraqui et al., 2017	Not specified	Morocco	Lower-middle	118,725	Hybrid	Non-diagnostic	Face-to-Face	N/A	Random

Dabbagh, 2017 a	Mixed	Iraq	Upper-middle	234,094	Authoritarian	Non-diagnostic	Self-report	N/A	Random
Roshanpajouh et al., 2019	Mixed	Iran	Upper-middle	445,345	Authoritarian	Non-diagnostic	Self-report	N/A	Random
Nikfarjam et al., 2016	Urban	Iran	Upper-middle	445,345	Authoritarian	Non-diagnostic	Self-report	N/A	Non-random
Stubbs et al., 2016	Not specified	Pakistan	Lower-middle	278,222	Hybrid	Diagnostic	Face-to-Face	DSM-IV	Random
	Not specified	Tunisia	Lower-middle	38,798	Flawed Democracy	Diagnostic	Face-to-Face	DSM-IV	Random
Veisani et al., 2017	Urban	Iran	Upper-middle	445,345	Authoritarian	Diagnostic	Face-to-Face	DSM-IV	Random
Moradinazar et al., 2020	Mixed	Iran	Upper-middle	445,345	Authoritarian	Non-diagnostic	Face-to-Face	N/A	Not specified
Asokan et al., 2019	Not specified	Bahrain	High	38,574	Authoritarian	Non-diagnostic	Face-to-Face	N/A	Convenience

Appendix 7: Sample size and main participant characteristics of papers included in the meta-analysis

Author, Year	Sample size	Education level	Income group	Population group	Age range	Mean age (years)	Males in the study sample (%)
El-Sherbiny et al., 2016	2219	Mixed	Mixed	General	>= 60 years	68.8	52.5%
Almuneef et al., 2016	9507	Mixed	Mixed	General	>= 18 years	34	52.1%
Baniasadi et al., 2019	1500	Mixed	Not specified	Disaster survivors	>=16 years	Not specified	52.5%
Gammouh et al., 2015	765	Mixed	Mixed	Disaster survivors	>= 18 years	Not specified	44.4%
Aly et al., 2018	1027	Not specified	Not specified	General	>= 60 years	Not specified	48.0%
Tayefi et al., 2017	9759	Not specified	Not specified	General	>= 35 years	48	40.0%
Alvi et al., 2017	624	Not specified	Mixed	General	>= 60 years	73	Not specified
Azizi et al., 2019	1520	Mixed	Not specified	General	>= 40 years	49.1	0.0%
Obeid et al., 2020	757	Mixed	Mixed	General	>= 18 years	30.3	54.8%
Hamdan-Mansour et al., 2017	1058	Mixed	Mixed	General	>= 60 years	68	54.3%
Aoun et al., 2018	450	Mixed	Mixed	Disaster survivors	>= 14 years	27.9	15.3%
Al Rashed et al., 2019	5172	>= Elementary	Mixed	General	>= 18 years	Not specified	41.0%
Farhood et al., 2016	991	Mixed	Mixed	General	>= 20 years	Not specified	47.3%
Alhassan et al., 2018	935	>= School	Mixed	General	>= 18 years	31.7	33.8%
Rafiey et al., 2019	600	Mixed	Mixed	Disaster survivors	>= 18 years	35.2	66.2%
Salah et al., 2015	1876	Mixed	Mixed	Disaster survivors	>= 18 years	35	44.3%
El-Gilany et al., 2018	474	Mixed	Not specified	General	I>= 60 years	67.3	49.8%
Bakhtiari et al., 2018	1560	Mixed	Not specified	General	>= 60 years	69.3	55.2%
Abou-Saleh et al., 2001	1388	Mixed	Not specified	General	>= 18 years	Not specified	50.9%
Mohammadi et al., 2005	25180	Mixed	Mixed	General	>= 18 years	Not specified	50.3%
Kim et al., 2007	1253	Not specified	Mixed	Disaster survivors	>=16 years	34	0.0%

De Jong et al., 2003	585	Mixed	Not specified	Disaster survivors	>=16 years	32	46.8%
Bhamani et al., 2013	953	Mixed	Mixed	General	>= 60 years	67	53.0%
Ghanem et al., 2009	14640	Mixed	Mixed	General	>= 18 years	Not specified	39.5%
Roberts et al., 2009	1242	Mixed	Not specified	General	>= 18 years	33	49.3%
Kadri et al., 2007	800	Mixed	Mixed	General	>= 15 years	32.2	50.0%
Nisar et al., 2004	1200	Not specified	Not specified	General	>= 18 years	Not specified	0.0%
Mufti et al., 2005	1301	Mixed	Mixed	General	>= 15 years	33.1	Not specified
El-Wasify et al., 2011	810	Mixed	Mixed	General	>= 15 years	38.9	64.0%
Alhasnawi et al., 2009	4332	Not specified	Not specified	General	>= 18 years	Not specified	Not specified
Ahmadvand et al., 2012	1740	Mixed	Not specified	General	>= 18 years	Not specified	Not specified
Kadri et al., 2010	5498	Mixed	Not specified	General	>= 15 years	Not specified	49.3%
Scholte et al., 2004	1010	Mixed	Not specified	General	>= 15 years	Not specified	46.4%
Karam et al., 2016	593	Mixed	Not specified	General	>= 60 years	Not specified	50.7%
Ruscio et al., 2017	4332	Not specified	Not specified	General	I>= 18 years	Not specified	Not specified
Mehrabi et al., 2019	1866	Mixed	Mixed	General	>= 18 years	34.8	55.9%
Khaled et al., 2020	1283	Mixed	Mixed	General	>= 18 years	Not specified	50.4%
Hasan et al., 2019	518	Mixed	Mixed	General	>= 18 years	Not specified	65.6%
Khazaie et al. 2019	2991	Mixed	Not specified	General	>= 15 years	27.02	44.4%
Degenhardt et al., 2019	1031	Mixed	Mixed	General	>= 18 years	Not specified	45.4%
Khaled et al., 2019	2520	Mixed	Mixed	General	>= 18 years	Not specified	80.1%
Kausar et al., 2015	1110	Mixed	Mixed	General	>= 13 years	Not specified	Not specified
Noorbala et al., 2015	35813	Mixed	Mixed	General	>= 15 years	Not specified	49.8%
Sharifi et al., 2017	4868	Mixed	Mixed	General	>= 15 years	21.8	59.5%
Stubbs et al., 2017	6501	Mixed	Mixed	General	>= 18 years	Not specified	Not specified
	5202	Mixed	Mixed	General	>= 18 years	Not specified	Not specified
	1183	Mixed	Mixed	General	>= 18 years	Not specified	Not specified

Rafiey et al., 2017	6027	Mixed	Mixed	General	>= 18 years	32	87.0%
Laraqui et al., 2017	1219	Mixed	Not specified	General	>= 20 years	37.9	100.0%
Dabbagh, 2017	3200	Mixed	Mixed	General	>= 18 years	Not specified	71.5%
Roshanpajouh et al., 2019	57450	Mixed	Mixed	General	>= 35 years	37.1	50.8%
Nikfarjam et al., 2016	7535	Mixed	Mixed	General	>= 18 years	30.8	48.2%
Stubbs et al., 2016	6097	Mixed	Mixed	General	>= 18 years	Not specified	Not specified
	4586	Mixed	Mixed	General	>= 18 years	Not specified	Not specified
Veisani et al., 2017	763	Mixed	Not specified	General	>= 15 years	37.9	44.6%
Moradinazar et al., 2020	130570	Mixed	Mixed	General	>= 35 years	49.7	44.5%
Asokan et al., 2019	571	Mixed	Mixed	General	>= 65 years	70	57.8%

Appendix 8: Analyses for depressive disorders

Appendix 8.1: Forest plot for depressive disorders

Author, year	Point estimate (95% CI)
Abou-Saleh et al. 2001	0.034 (0.026 – 0.045)
Nisar et al. 2004	0.076 (0.062 – 0.092)
Scholte et al. 2004	0.387 (0.358 – 0.418)
Mohammadi et al. 2005	0.030 (0.028 – 0.032)
Mohammadi et al. 2005	0.003 (0.003 – 0.004)
Mufti et al. 2005	0.279 (0.254 – 0.306)
Kim et al. 2007	0.311 (0.286 – 0.337)
Alhasnawi et al. 2009	0.072 (0.065 – 0.080)
Ghanem et al. 2009	0.027 (0.024 – 0.030)
Roberts et al. 2009	0.499 (0.471 – 0.527)
Kadri et al. 2010	0.265 (0.254 – 0.277)
El-Wasify et al. 2011	0.015 (0.008 – 0.026)
Ahmadvand et al. 2012	0.081 (0.060 – 0.108)
Ahmadvand et al. 2012	0.053 (0.037 – 0.077)
Ahmadvand et al. 2012	0.008 (0.003 – 0.021)
Bhamani et al. 2013	0.406 (0.375 – 0.438)
Gammouh et al. 2015	0.295 (0.264 – 0.329)
Kausar et al. 2015	0.053 (0.041 – 0.068)
Kausar et al. 2015	0.034 (0.025 – 0.047)
Noorbala et al. 2015	0.104 (0.101 – 0.107)
Almuneef et al. 2016	0.127 (0.120 – 0.134)
El-Sherbiny et al. 2016	0.745 (0.726 – 0.763)
Farhood et al. 2016	0.058 (0.045 – 0.074)
Karam et al. 2016	0.084 (0.064 – 0.110)
Stubbs et al. 2016	0.074 (0.067 – 0.082)
Stubbs et al. 2016	0.064 (0.058 – 0.070)
Alvi et al. 2017	0.223 (0.192 – 0.257)
Hamdan-Mansour et al. 2017	0.445 (0.415 – 0.475)
Tayefi et al. 2017	0.348 (0.339 – 0.358)
Veisani et al. 2017	0.203 (0.176 – 0.233)
Alhassan et al. 2018	0.230 (0.204 – 0.258)
Aly et al. 2018	0.627 (0.597 – 0.656)
Bakhtiari et al. 2018	0.426 (0.402 – 0.451)
El-Gilany et al. 2018	0.449 (0.405 – 0.494)
Al Rasheed et al. 2019	0.086 (0.079 – 0.094)
Asokan et al. 2019	0.089 (0.069 – 0.116)
Azizi et al. 2019	0.110 (0.095 – 0.127)
Baniasadi et al. 2019	0.399 (0.374 – 0.424)
Khalid et al. 2019	0.496 (0.476 – 0.515)
Khazaie et al. 2019	0.216 (0.202 – 0.231)
Obeid et al. 2020	0.597 (0.562 – 0.631)
	0.148 (0.107 – 0.201)

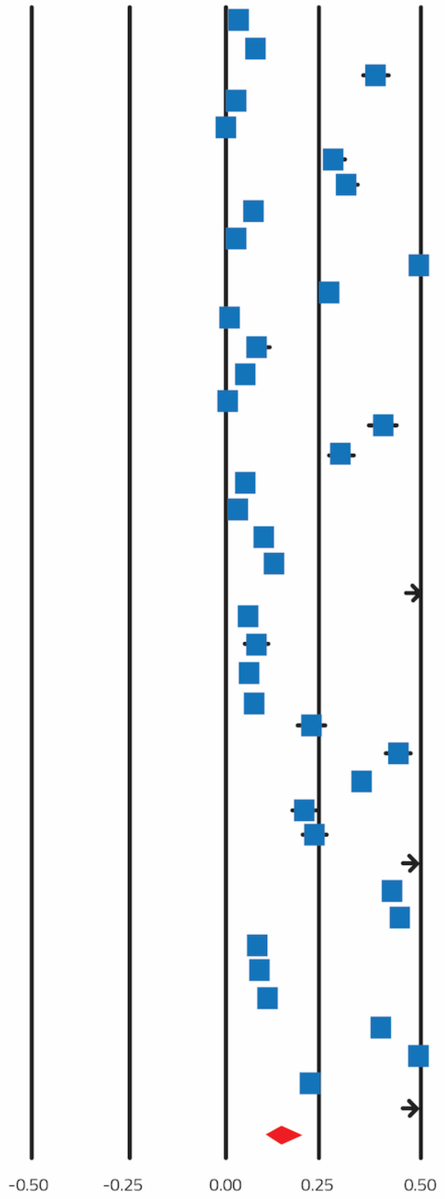

Appendix 8.2: Funnel plot for depressive disorders

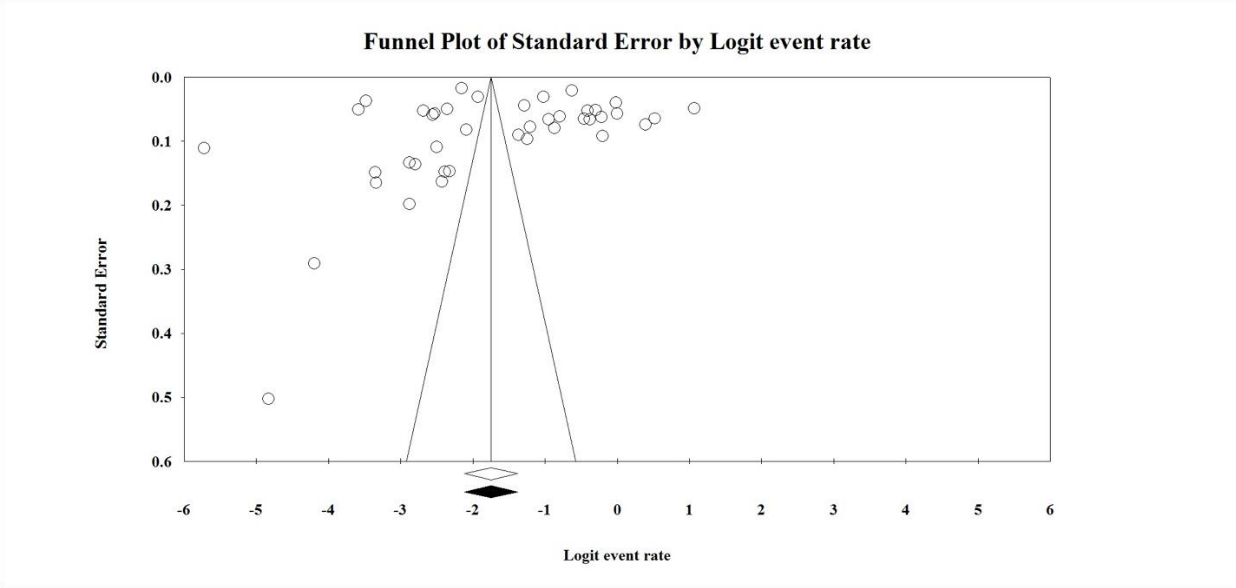

Appendix 8.3: Random-effects subgroup analysis of prevalence of depressive disorders stratified by potential determinants and methodological variables

	Point estimates and 95% CI							
	No. of cohorts	Prevalence (%)	Lower limit	Upper limit	I^2 (%)	Q-value	df (Q)	*p*-value
**EMR Countries**								
Afghanistan	2	33.0	7.0	75.0	96.47	12.82	13.00	0.46
Bahrain	1	9.0	1.0	56.0	0.00			
Egypt	5	22.0	8.0	47.0	99.92			
Iran	12	9.0	5.0	18.0	99.87			
Iraq	1	7.0	1.0	50.0	0.00			
Jordan	2	37.0	9.0	78.0	97.60			
Lebanon	3	17.0	4.0	47.0	99.66			
Morocco	1	27.0	3.0	82.0	0.00			
Pakistan	6	10.0	4.0	25.0	99.48			
Qatar	1	50.0	7.0	93.0	0.00			
Saudi Arabia	3	14.0	4.0	41.0	98.74			
Sudan	2	40.0	10.0	80.0	98.89			
Tunisia	1	7.0	1.0	51.0	0.00			
UAE	1	3.0	0.0	32.0	0.00			
**Type of population**								

Disaster survivors	3	33.0	11.0	66.0	94.04	2.51	1.00	0.11
General	38	14.0	10.0	19.0	99.82			
**Type of regime**								
Authoritarian	30	16.0	11.0	23.0	99.84	0.60	2.00	0.74
Flawed Democracy	1	7.0	1.0	47.0	0.00			
Hybrid	10	13.0	7.0	25.0	99.57			
**Type of screening method**								
Diagnostic	24	8.0	5.0	12.0	99.72	21.95	1.00	<0.01
Non-diagnostic	17	32.0	21.0	45.0	99.83			
**Country's income group**								
High	6	13.0	5.0	30.0	99.76	2.67	3.00	0.44
Low	5	28.0	11.0	54.0	99.12			
Lower-middle	12	16.0	8.0	27.0	99.85			
Upper-middle	18	12.0	7.0	20.0	99.83			

Appendix 8.4: Meta-regression for depressive disorders by mean-age and GDP

	**Coefficient**	**Standard Error**	**95% Lower limit**	**95% Upper limit**	**Z-value**	***p*-value**
**Model 1**						
Intercept	-1.68	0.79	-3.23	-0.13	-2.12	0.03
Age	0.01	0.02	-0.02	0.04	0.95	0.34
**Model 2**						
Intercept	-1.23	0.39	-1.99	-0.47	-3.16	<0.01
GDP	< 0.0	0.0	< 0.0	0.0	-1.64	0.10

Appendix 9: Analyses for bipolar disorders

Appendix 9.1: Forest plot for bipolar disorders

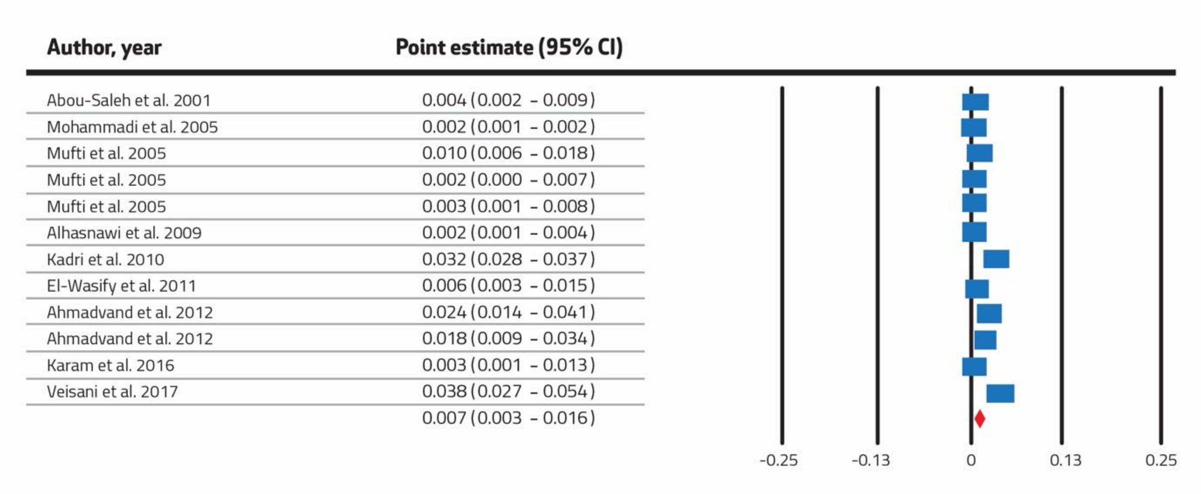

Appendix 9.2: Funnel plot for bipolar disorders

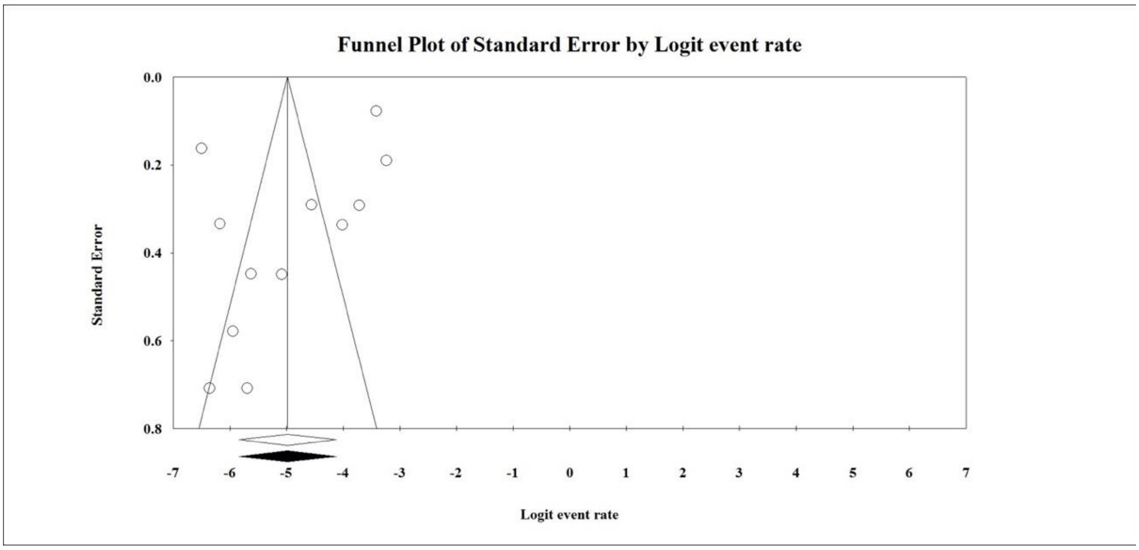

Appendix 9.3: Random-effects subgroup analysis of prevalence bipolar disorders stratified by potential determinants

	Point estimates and 95% CI							
	**No. of cohorts**	**Prevalence (%)**	**Lower limit**	**Upper limit**	**I^2 (%)**	**Q-value**	**df (Q)**	***p*-value**
**EMR Countries**								
Afghanistan	3	0.0	0.0	3.0	77.04	2.34	6.00	0.89
Egypt	1	1.0	0.0	17.0	0.00			
Iran	4	1.0	0.0	7.0	98.48			
Iraq	1	0.0	0.0	6.0	0.00			
Lebanon	1	0.0	0.0	12.0	0.00			

Morocco	1	3.0	0.0	50.0	0.00			
UAE	1	0.0	0.0	11.0	0.00			
**Type of regime**								
Authoritarian	10	1.0	0.0	2.0	95.97	0.30	1.00	0.58
Hybrid	2	1.0	0.0	9.0	90.23			
**Country's income group**								
High	1	0.0	0.0	9.0	0.00	0.99	3.00	0.80
Low	3	0.0	0.0	3.0	77.04			
Lower-middle	2	1.0	0.0	12.0	92.59			
Upper-middle	6	1.0	0.0	3.0	97.65			

Appendix 9.4: Meta-regression for bipolar disorders by mean-age and GDP

	**Coefficient**	**Standard Error**	**95% Lower limit**	**95% Upper limit**	**Z-value**	***p*-value**
**Model 1**						
Intercept	-13.20	5.68	-24.33	-2.08	-2.33	0.02
Age	0.24	0.16	-0.08	0.55	1.48	0.14
**Model 2**						
Intercept	-5.37	0.59	-6.52	-4.22	-9.16	0.00
GDP	0.00	0.00	< 0.0	0.00	0.87	0.38

Appendix 10: Analyses for generalised anxiety disorders

Appendix 10.1: Forest plot for generalised anxiety disorders

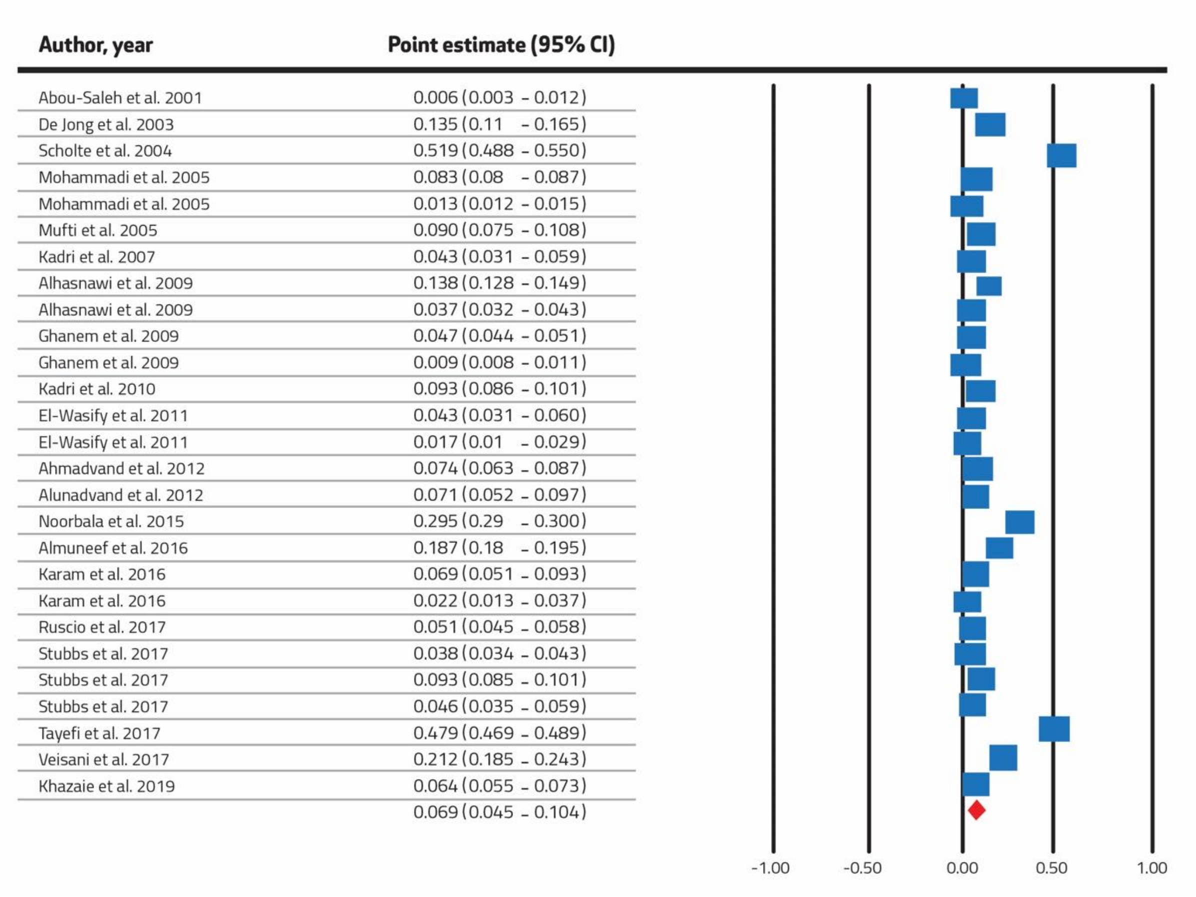

Author, year	Point estimate (95% CI)
Abou-Saleh et al. 2001	0.006 (0.003 - 0.012)
De Jong et al. 2003	0.135 (0.11 - 0.165)
Scholte et al. 2004	0.519 (0.488 - 0.550)
Mohammadi et al. 2005	0.083 (0.08 - 0.087)
Mohammadi et al. 2005	0.013 (0.012 - 0.015)
Mufti et al. 2005	0.090 (0.075 - 0.108)
Kadri et al. 2007	0.043 (0.031 - 0.059)
Alhasnawi et al. 2009	0.138 (0.128 - 0.149)
Alhasnawi et al. 2009	0.037 (0.032 - 0.043)
Ghanem et al. 2009	0.047 (0.044 - 0.051)
Ghanem et al. 2009	0.009 (0.008 - 0.011)
Kadri et al. 2010	0.093 (0.086 - 0.101)
El-Wasify et al. 2011	0.043 (0.031 - 0.060)
El-Wasify et al. 2011	0.017 (0.01 - 0.029)
Ahmadvand et al. 2012	0.074 (0.063 - 0.087)
Alunadvand et al. 2012	0.071 (0.052 - 0.097)
Noorbala et al. 2015	0.295 (0.29 - 0.300)
Almuneef et al. 2016	0.187 (0.18 - 0.195)
Karam et al. 2016	0.069 (0.051 - 0.093)
Karam et al. 2016	0.022 (0.013 - 0.037)
Ruscio et al. 2017	0.051 (0.045 - 0.058)
Stubbs et al. 2017	0.038 (0.034 - 0.043)
Stubbs et al. 2017	0.093 (0.085 - 0.101)
Stubbs et al. 2017	0.046 (0.035 - 0.059)
Tayefi et al. 2017	0.479 (0.469 - 0.489)
Veisani et al. 2017	0.212 (0.185 - 0.243)
Khazaie et al. 2019	0.064 (0.055 - 0.073)
	0.069 (0.045 - 0.104)

Appendix 10.2: Funnel plot for generalised anxiety disorders

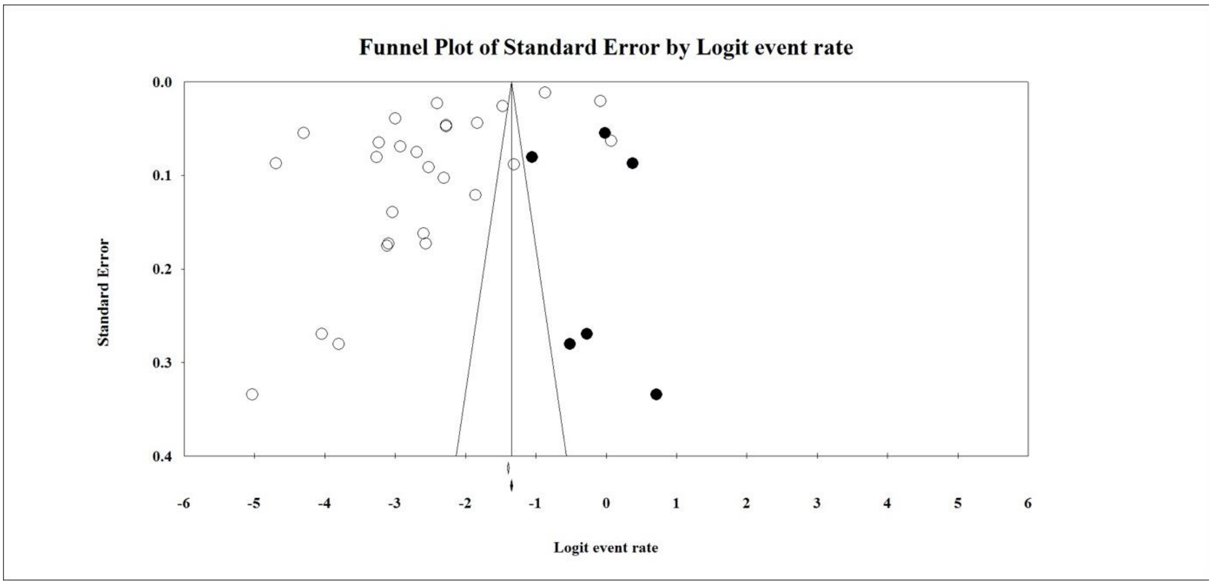

Appendix 10.3: Random-effects subgroup analysis of prevalence of generalised anxiety disorders stratified by potential determinants and methodological variables

	Point estimates and 95% CI							
	**No. of cohorts**	**Prevalence (%)**	**Lower limit**	**Upper limit**	**I^2 (%)**	**Q-value**	**df (Q)**	***p*-value**
**EMR Countries**								
Afghanistan	2	25.0%	6.0%	62.0%	99.74	14.99	11	0.18
Egypt	4	2.0%	1.0%	7.0%	99.07			
Iran	8	11.0%	5.0%	22.0%	99.93			
Iraq	3	6.0%	2.0%	20.0%	99.4			
Lebanon	2	4.0%	1.0%	17.0%	92.7			
Morocco	2	6.0%	1.0%	25.0%	95.3			
Pakistan	1	4.0%	0.0%	27.0%	0.00			
Palestine	1	14.0%	2.0%	60.0%	0.00			
Saudi Arabia	1	19.0%	2.0%	69.0%	0.00			
Tunisia	1	9.0%	1.0%	50.0%	0.00			
UAE	2	2.0%	0.0%	9.0%	96.69			
**Type of population**								
Disaster survivors	1	14.0%	2.0%	61.0%	0.00	0.41	1	0.52
General	26	7.0%	4.0%	10.0%	99.87			
**Type of regime**								
Authoritarian	21	7.0%	5.0%	12.0%	99.88	0.74	2	0.69
Flawed Democracy	1	9.0%	1.0%	50.0%	0.00			
Hybrid	5	5.0	2.0	12.0	97.62			
**Type of screening method**								
Diagnostic	22	6.0%	4.0%	9.0%	99.62	3.37	1	0.07
Non-diagnostic	5	13.0%	6.0%	26.0%	99.9			
**Country's income group**								
High	3	4.0%	1.0%	14.0%	99.14	5.95	3	0.11
Low	2	25.0%	6.0%	61.0%	99.74			
Lower-middle	9	5.0%	2.0%	9.0%	99.09			
Upper-middle	13	8.0%	5.0%	15.0%	99.9			

Appendix 10.4: Meta-regression for generalised anxiety disorders by mean-age and GDP

	Coefficient	Standard Error	95% Lower limit	95% Upper limit	Z-value	*p*-value
**Model 1**						
Intercept	-5.31	2.09	-9.41	-1.22	-2.54	0.01
Age	0.09	0.06	-0.03	0.20	1.51	0.13
**Model 2**						
Intercept	-2.61	0.43	-3.44	-1.77	-6.12	0.00
GDP	-0.00	0.00	-0.00	0.00	-0.46	0.98

Appendix 11: Analyses for post-traumatic stress disorder

Appendix 11.1: Forest plot for post-traumatic stress disorder

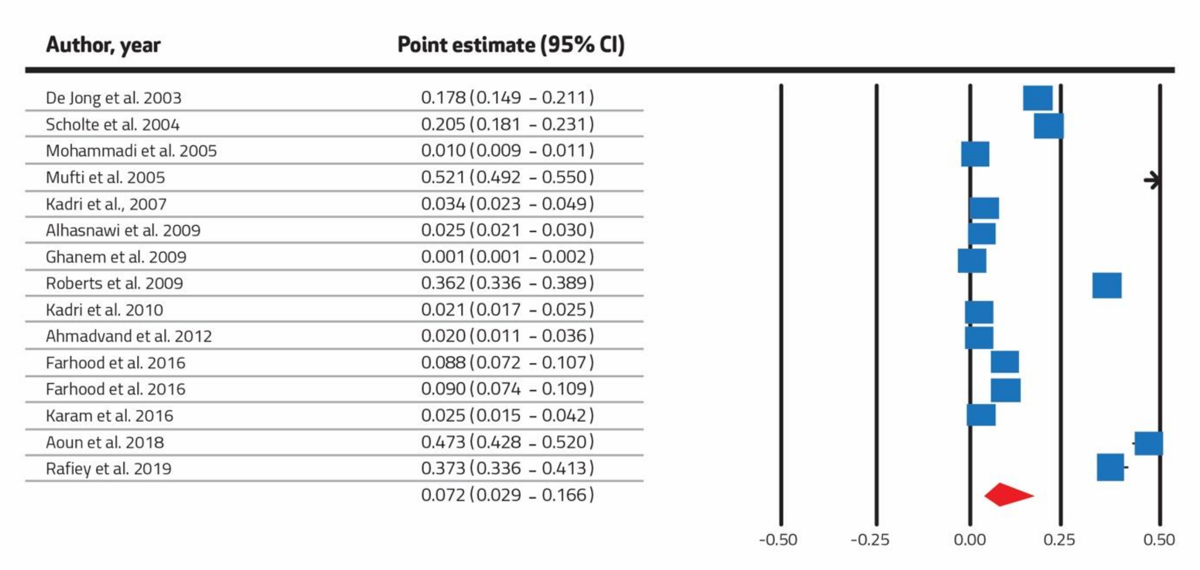

Appendix 11.2: Funnel plot for post-traumatic stress disorder

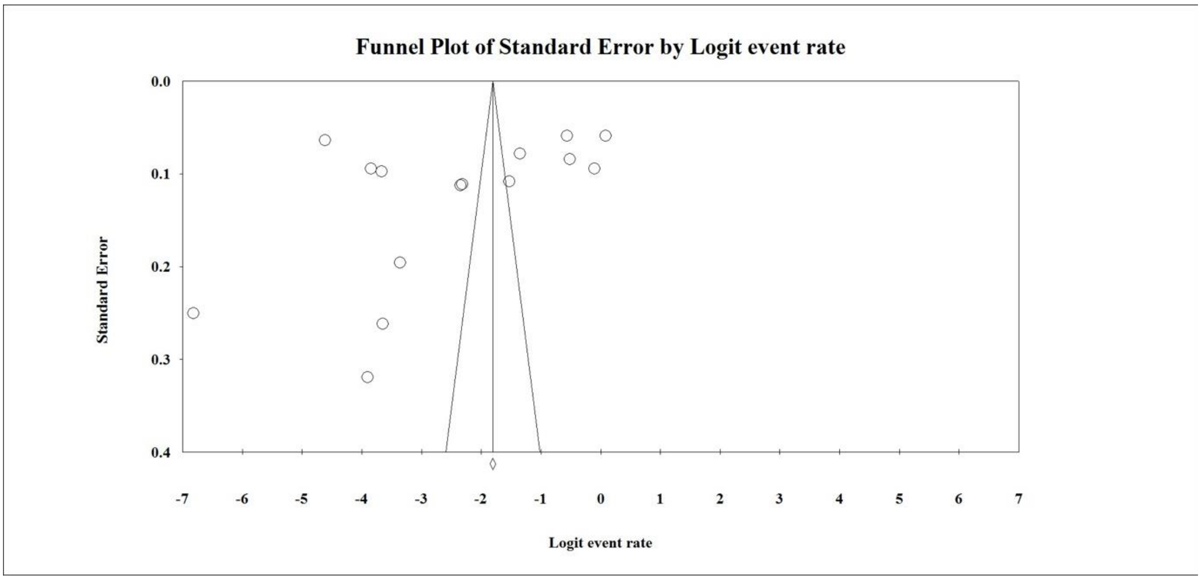

Appendix 11.3: Random-effects subgroup analysis of prevalence of post-traumatic stress disorder stratified by potential determinants and methodological variables

	Point estimates and 95% CI							
	**No. of cohorts**	**Prevalence (%)**	**Lower limit**	**Upper limit**	**I^2 (%)**	**Q-value**	**df (Q)**	***p*-value**
**EMR Countries**								
Afghanistan	2	35.0%	4.0%	87.0%	99.54	1.66	7	0.15
Egypt	1	0.0%	0.0%	4.0%	0.00			
Iran	3	5.0%	1.0%	28.0%	99.87			
Iraq	1	2.0%	0.0%	47.0%	0.00			
Lebanon	4	11.0%	2.0%	42.0%	99.24			
Morocco	2	3.0%	0.0%	25.0%	80.47			
Palestine	1	18.0%	1.0%	88.0%	0.00			
Sudan	1	36.0%	2.0%	95.0%	0.00			
**Type of population**								
Disaster survivors	3	33.0%	6.0%	80.0%	98.04	3.77	1	0.05
General	12	5.0%	2.0%	12.0%	99.76			
**Type of regime**								
Authoritarian	9	7.0%	2.0%	22.0%	99.8	0.01	1	0.95
Hybrid	6	7.0%	2.0%	26.0%	99.42			
**Type of screening method**								
Diagnostic	12	6.0%	2.0%	15.0%	99.78	0.97	1	0.33
Non-diagnostic	3	17.0%	3.0%	61.0%	99.39			
**Country's income group**								
Low	3	35.0%	8.0%	76.0%	98.09	7.33	2	0.03
Lower-middle	4	2.0%	0.0%	9.0%	99.39			
Upper-middle	8	7.0%	2.0%	18.0%	99.72			

Appendix 11.4: Meta-regression for post-traumatic stress disorder by mean-age and GDP

	**Coefficient**	**Standard Error**	**95% Lower limit**	**95% Upper limit**	**Z-value**	***p*-value**
**Model 1**						
Intercept	0.08	6.92	-13.49	13.64	0.02	0.99
Age	-0.03	0.21	-0.45	0.39	-0.15	0.88
**Model 2**						
Intercept	-1.7	0.6	-2.86	-0.53	-2.85	<0.01
GDP	0.00	0.00	0.00	0.00	-2.26	0.04

Appendix 12: Analyses for obsessive-compulsive disorder

Appendix 12.1: Forest plot for obsessive-compulsive disorder

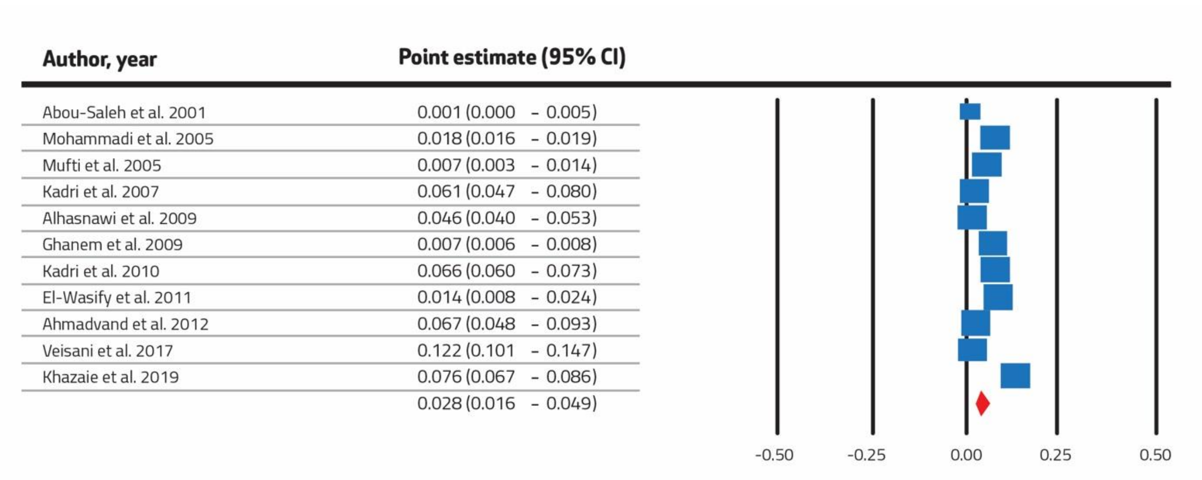

Appendix 12.2: Funnel plot for obsessive-compulsive disorder

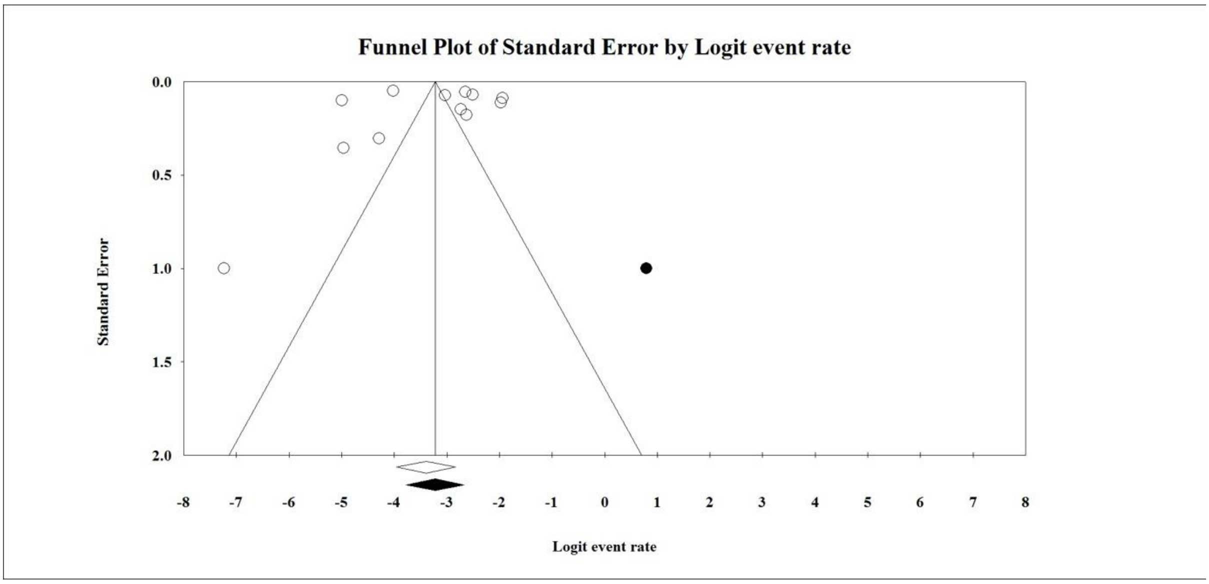

Appendix 12.3: Random-effects subgroup analysis of prevalence of obsessive-compulsive disorder stratified by potential determinants

	Point estimates and 95% CI							
	**No. of cohorts**	**Prevalence (%)**	**Lower limit**	**Upper limit**	**I^2 (%)**	**Q-value**	**df (Q)**	***p*-value**
**EMR Countries**								
Afghanistan	1	1.0%	0.0%	5.0%	0.0	15.45	6	0.01
Egypt	2	1.0%	0.0%	4.0%	79.36			
Iran	4	6.0%	2.0%	14.0%	99.42			
Iraq	1	5.0%	1.0%	25.0%	0.0			
Morocco	2	6.0%	2.0%	21.0%	0.0			
UAE	1	0.0%	0.0%	1.0%	0.0			
**Type of regime**								
Authoritarian	9	2.0%	1.0%	4.0%	99.04	1.88	1	0.17
Hybrid	2	6.0%	2.0%	21.0%	0.0			
**Country's income group**								
High	1	0.1%	0.0%	1.0%	0.0	10.58	3	0.01
Low	1	0.7%	0.1%	6.0%	0.0			
Lower-middle	4	3.0%	1.0%	7.0%	99.32			
Upper-middle	5	5.0%	2.0%	13.0%	99.24			

Appendix 12.4: Meta-regression for obsessive-compulsive disorder by mean-age and GDP

	**Coefficient**	**Standard Error**	**95% Lower limit**	**95% Upper limit**	**Z-value**	***p*-value**
**Model 1**						
Intercept	-1.55	3.85	-9.11	6.00	-0.40	0.69
Age	-0.05	0.11	-0.27	0.17	-0.44	0.66
**Model 2**						
Intercept	-3.83	0.83	-5.44	-2.21	-4.63	0.00
GDP	0.00	0.00	0.00	0.00	-0.33	0.74

Appendix 13: Analyses for phobic disorders

Appendix 13.1: Forest plot for phobic disorders

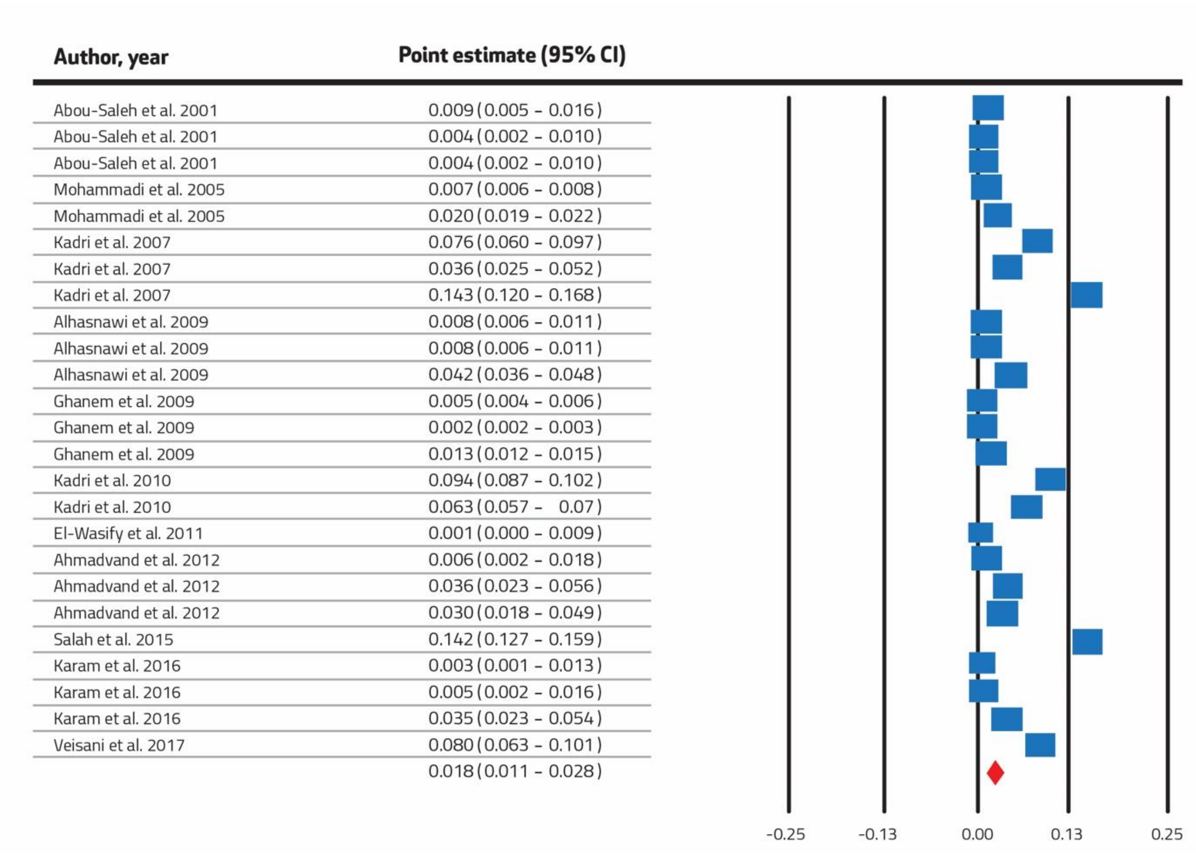

Appendix 13.2: Funnel plot for phobic disorders

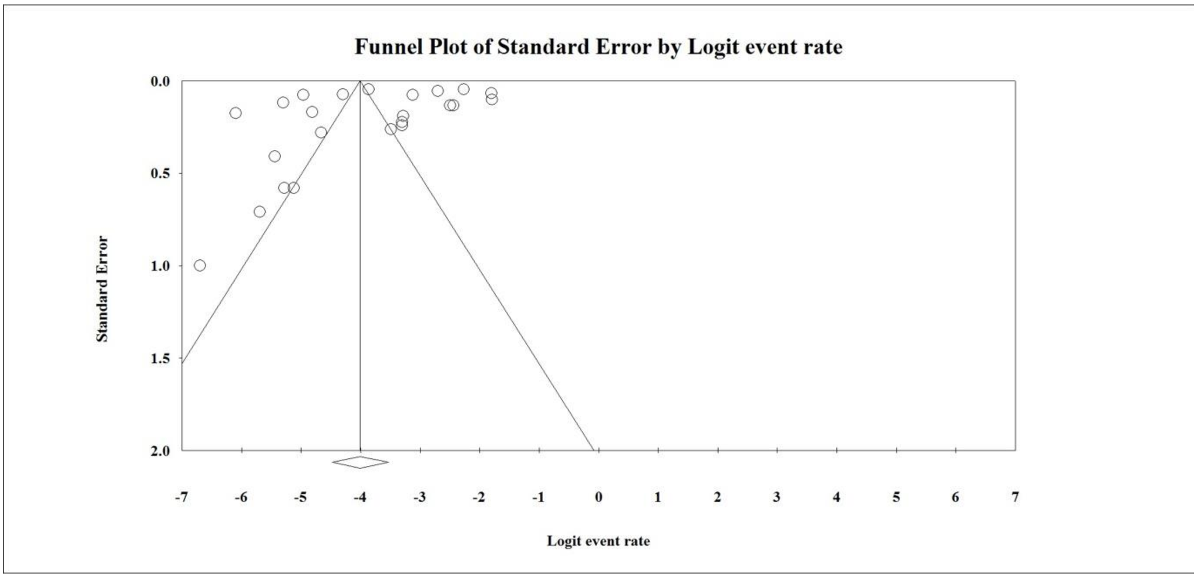

Appendix 13.3: Random-effects subgroup analysis of prevalence of phobic disorders stratified by potential determinants

	Point estimates and 95% CI							
	**No. of cohorts**	**Prevalence (%)**	**Lower limit**	**Upper limit**	**I^2 (%)**	**Q-value**	**df (Q)**	***p*-value**
**EMR Countries**								
Egypt	4	0.0%	0.0%	1.0%	97.6	49.2	6.0	<0.01
Iran	6	2.0%	1.0%	4.0%	98.45			
Iraq	3	1.0%	1.0%	3.0%	98.58			
Lebanon	3	1.0%	0.0%	3.0%	89.19			
Morocco	5	8.0%	4.0%	13.0%	95.82			
Sudan	1	14.0%	4.0%	39.0%	0.00			
UAE	3	1.0%	0.0%	1.0%	46.72			
**Type of population**								
Disaster survivors	1	14.0%	2.0%	60.0%	0.00	4.05	1.0	0.04
General	24	2.0%	1.0%	3.0%	99.1			
**Type of regime**								
Authoritarian	17	1.0%	1.0%	2.0%	99.14	6.7	1.0	0.01
Hybrid	8	4.0%	2.0%	8.0%	95.55			
**Country's income group**								
High	3	1.0%	0.0%	2.0%	46.72	8.11	3.0	<0.01
Low	1	14.0%	2.0%	58.0%	0.00			
Lower-middle	9	2.0%	1.0%	5.0%	99.46			
Upper-middle	12	2.0%	1.0%	3.0%	97.84			

Appendix 13.4: Meta-regression for phobic disorders by mean-age and GDP

	**Coefficient**	**Standard Error**	**95% Lower limit**	**95% Upper limit**	**Z-value**	***p*-value**
**Model 1**						
Intercept	5.77	6.00	-5.99	17.53	0.96	0.34
Age	-0.25	0.17	-0.59	0.10	-1.43	0.15
**Model 2**						
Intercept	-3.13	0.49	-4.09	-2.16	-6.36	<0.01
GDP	0.00	0.00	0.00	0.00	-2.06	0.04

Appendix 14: Analyses for panic disorders

Appendix 14.1: Forest plot for panic disorders

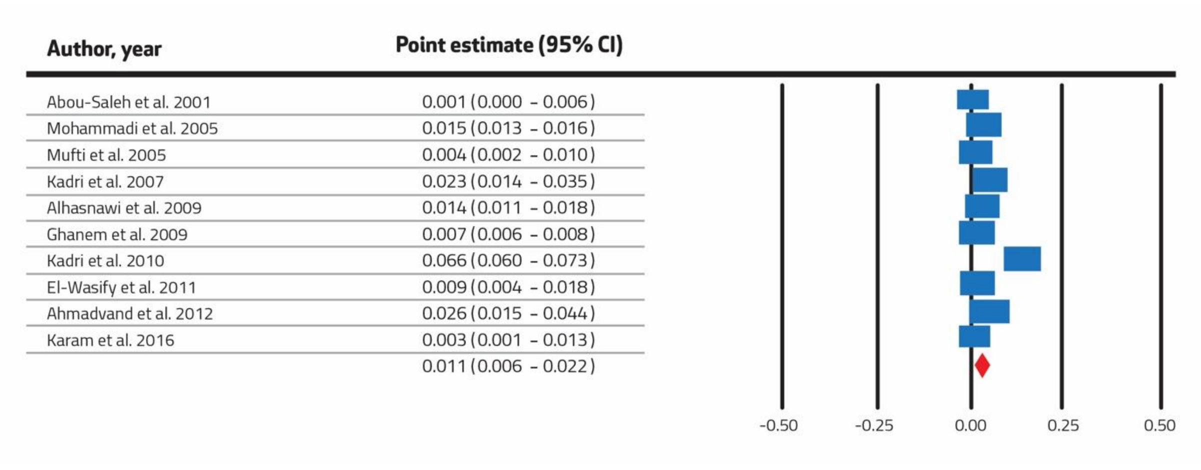

Appendix 14.2: Funnel plot for panic disorders

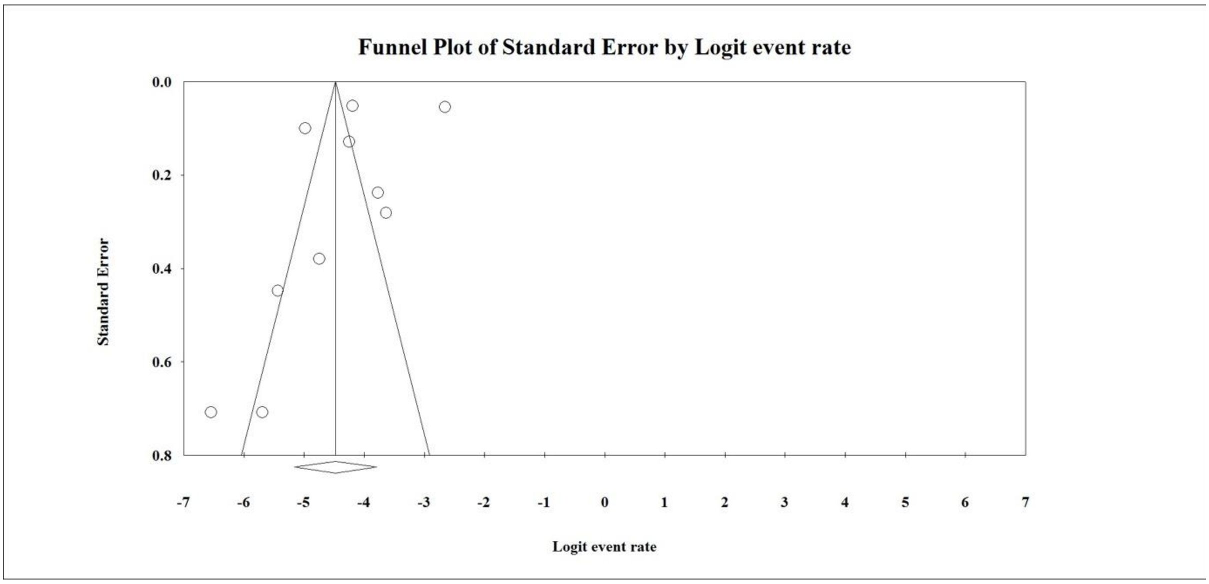

Appendix 14.3: Random-effects subgroup analysis of prevalence of panic disorders stratified by potential determinants

	Point estimates and 95% CI							
	**No. of cohorts**	**Prevalence (%)**	**Lower limit**	**Upper limit**	**I^2 (%)**	**Q-value**	**df (Q)**	***p*-value**
**EMR Countries**								
Afghanistan	1	0.4%	0.1%	2.0%	0.00	21.02	7.00	<0.01
Egypt	2	1.0%	0.0%	2.0%	0.00			
Iran	2	2.0%	1.0%	5.0%	74.06			

Iraq	1	1.0%	0.0%	5.0%	0.00			
Lebanon	1	0.0%	0.0%	2.0%	0.00			
Morocco	2	4.0%	2.0%	10.0%	95.25			
UAE	1	0.0%	0.0%	1.0%	0.00			
**Type of regime**								
Authoritarian	7	1.0%	1.0%	2.0%	91.65	5.30	1.00	0.02
Hybrid	3	3.0%	1.0%	5.0%	94.83			
**Country's income group**								
High	1	0.0%	0.0%	2.0%	0.00	3.46	3.00	0.33
Low	1	0.0%	2.0%	12.0%	0.00			
Lower-middle	4	2.0%	1.0%	5.0%	99.32			
Upper-middle	4	1.0%	0.0%	4.0%	65.16			

Appendix 14.4: Meta-regression for panic disorders by GDP

	Coefficient	Standard Error	95% Lower limit	95% Upper limit	Z-value	*p*-value
**Model 1**						
Intercept	-4.39	0.61	-5.58	-3.19	-7.17	0.00
GDP	-0.00	0.00	-0.00	0.00	-1.39	0.16

Appendix 15: Analyses for substance use

Appendix 15.1: Forest plot for substance use

Author, year	Point estimate (95% CI)
Abou-Saleh et al. 2001	0.004 (0.002 – 0.010)
Mufti et al. 2005	0.001 (0.000 – 0.006)
Mufti et al. 2005	0.002 (0.000 – 0.007)
Mufti et al. 2005	0.003 (0.001 – 0.008)
Alhasnawi et al. 2009	0.007 (0.005 – 0.010)
Alhasnawi et al. 2009	0.000 (0.000 – 0.002)
Alhasnawi et al. 2009	0.002 (0.001 – 0.004)
Alhasnawi et al. 2009	0.009 (0.007 – 0.012)
Alhasnawi et al.2009	0.002 (0.001 – 0.004)
Ghanem et al. 2009	0.000 (0.000 – 0.001)
Ghanem et al. 2009	0.001 (0.001 – 0.002)
Kadri et al. 2010	0.034 (0.030 – 0.039)
Kadri et al. 2010	0.058 (0.052 – 0.065)
Almuneef et al. 2016	0.085 (0.080 – 0.091)
Almuneef et al. 2016	0.080 (0.074 – 0.085)
Almuneef et al. 2016	0.375 (0.365 – 0.385)
Karam et al. 2016	0.001 (0.000 – 0.013)
Lien et al. 2016	0.142 (0.113 – 0.177)
Nikfarjam et al. 2016	0.005 (0.003 – 0.006)
Nikfarjam et al. 2016	0.003 (0.002 – 0.005)
Nikfarjam et al. 2016	0.015 (0.012 – 0.018)
Nikfarjam et al. 2016	0.003 (0.002 – 0.005)
Nikfatjam et al. 2016	0.006 (0.004 – 0.008)
Nikfatjam et al. 2016	0.003 (0.002 – 0.004)
Dabbagh 2017	0.081 (0.072 – 0.091)
Dabbagh, 2017	0.220 (0.206 – 0.235)
Dabbagh. 2017	0.064 (0.056 – 0.073)
Laraqui et al. 2017	0.365 (0.338 – 0.392)
Laraqui et al. 2017	0.362 (0.335 – 0.389)
Laraqui et al. 2017	0.062 (0.050 – 0.077)
Laraqui et al. 2017	0.093 (0.078 – 0.110)
Laraqui et al. 2017	0.585 (0.557 – 0.612)
Laraqui et al. 2017	0.123 (0.106 – 0.143)
Rafiey et al. 2017	0.043 (0.038 – 0.049)
Sharifi et al. 2017	0.075 (0.068 – 0.083)
Degenhardt et al. 2019	0.062 (0.049 – 0.079)
Hasan et al. 2019	0.324 (0.285 – 0.366)
Khaled et al. 2019	0.292 (0.268 – 0.318)
Khazaie et al. 2019	0.024 (0.019 – 0.031)
Khazaie et al. 2019	0.021 (0.017 – 0.027)
Khazaie et al. 2019	0.046 (0.039 – 0.054)
Khazaie et al. 2019	0.007 (0.005 – 0.011)
Khazaie et al. 2019	0.002 (0.001 – 0.004)
Khazaie et al. 2019	0.002 (0.001 – 0.004)
Khazaie et al. 2019	0.051 (0.044 – 0.059)
Khazaie et al. 2019	0.009 (0.006 – 0.014)
Khazaie et al. 2019	0.002 (0.001 – 0.004)
Khazaie et al. 2019	0.000 (0.000 – 0.003)
Mehrabi et al. 2019	0.289 (0.269.– 0.310)
Mehrabi et al. 2019	0.148 (0.132 – 0.165)
Mehrabi et al. 2019	0.081 (0.069 – 0.094)
Roshanpajouh et al. 2019	0.085 (0.083 – 0.087)
Roshanpajouh et al. 2019	0.004 (0.004 – 0.005)
Moradinazar et al. 2020	0.477 (0.474 – 0.480)
Moradinazar et al. 2020	0.111 (0.109 – 0.113)
Moradinazar et al. 2020	0.340 (0.337 – 0.343)
Moradinazar et al. 2020	0.433 (0.430 – 0.436)
Roshanpajouh et al. 2020	0.033 (0.032 – 0.034)
	0.031 (0.023 – 0.040)

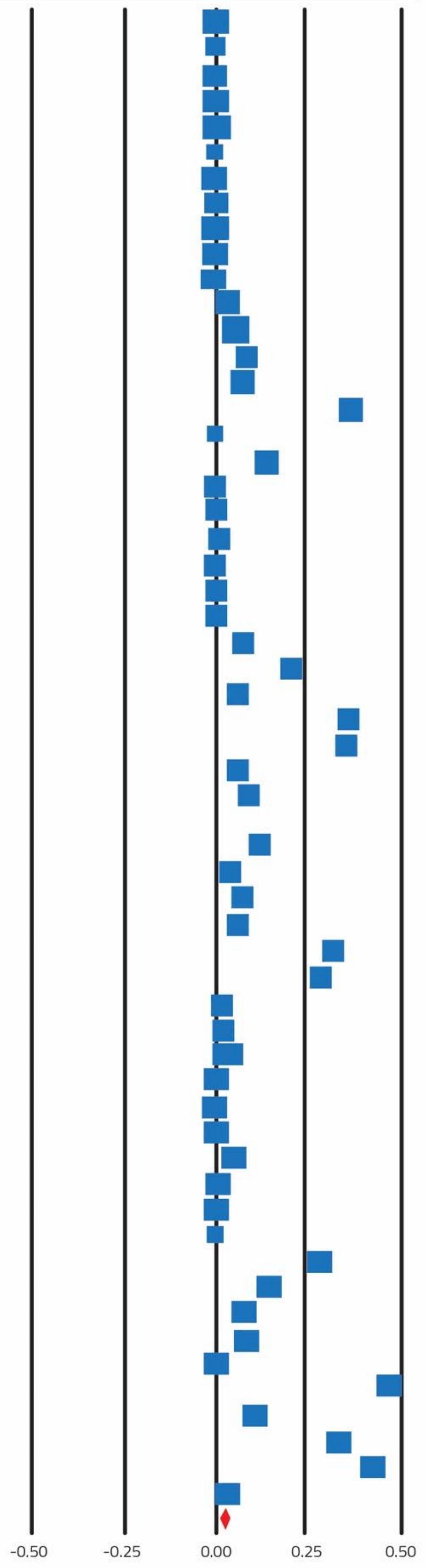

Appendix 15.2: Funnel plot for substance use

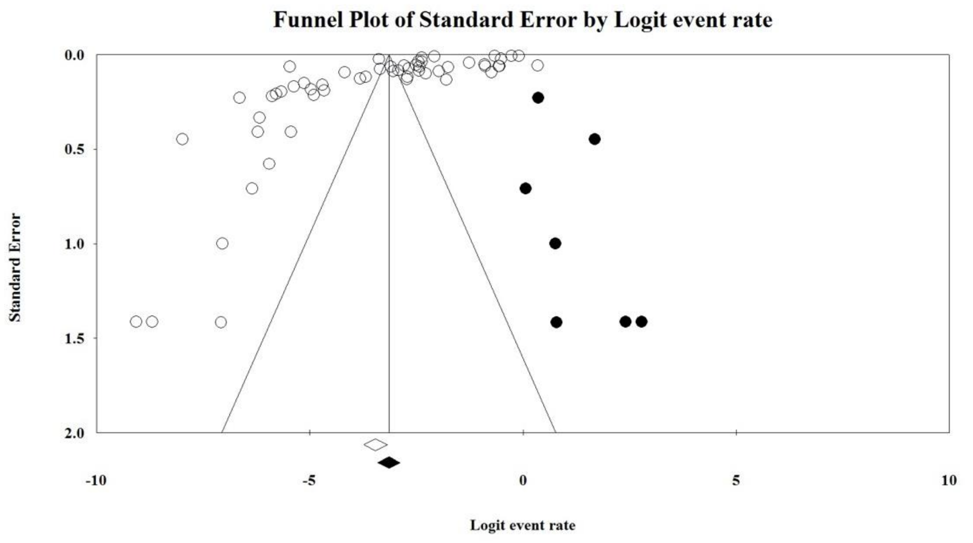

Appendix 15.3: Random-effects subgroup analysis of prevalence of substance use stratified by potential determinants and methodological factors

	Point estimates and 95% CI							
	**No. of cohorts**	**Prevalence (%)**	**Lower limit**	**Upper limit**	**I^2 (%)**	**Q-value**	**df (Q)**	***p*-value**
**EMR Countries**								
Afghanistan	3	3	0.0%	0.0%	1.0%	0.00	99.56	<0.01
Egypt	2	2	0.0%	0.0%	0.0%	85.84		
Iran	28	28	3.0%	2.0%	4.0%	99.97		
Iraq	8	8	1.0%	1.0%	3.0%	99.46		
Lebanon	2	2	2.0%	0.0%	11.0%	89.40		
Morocco	8	8	15.0%	8.0%	26.0%	99.75		
Pakistan	1	1	32.0%	6.0%	78.0%	0.00		
Qatar	1	1	29.0%	5.0%	75.0%	0.00		
Saudi Arabia	3	3	14.0%	5.0%	34.0%	99.94		
UAE	1	1	0.0%	0.0%	4.0%	0.00		
**Type of regime**								
Authoritarian	46	2.0%	2.0%	3.0%	99.95	31.46	1.00	<0.01
Hybrid	11	13.0%	7.0%	22.0%	99.66			
**Type of screening method**								
Diagnostic	27	1.0%	0.5%	1.2%	98.99	77.02	1.00	<0.01
Non-diagnostic	30	8.0%	6.0%	11.0%	99.97			
**Country's income group**								
High	5	10.0%	4.0%	21.0%	99.88	34.98	3.00	<0.01
Low	3	0.2%	0.0%	1.0%	0.00			

Lower-middle	11	7.0%	4.0%	12.0%	99.72
Upper-middle	38	2.0%	2.0%	3.0%	99.96

Appendix 15.4: Meta-regression for substance use by mean-age and GDP

	Coefficient	Standard Error	95% Lower limit	95% Upper limit	Z-value	*p*-value
**Model 1**						
Intercept	-9.9	1.36	-12.56	-7.24	-7.3	<0.01
Age	0.19	0.04	0.12	0.26	4.9	<0.01
**Model 2**						
Intercept	-4.01	0.63	-5.24	-2.77	-6.37	0.0
GDP	0.00	0.00	0.00	0.00	0.60	0.55

Appendix 15.5: Forest plot for tobacco use

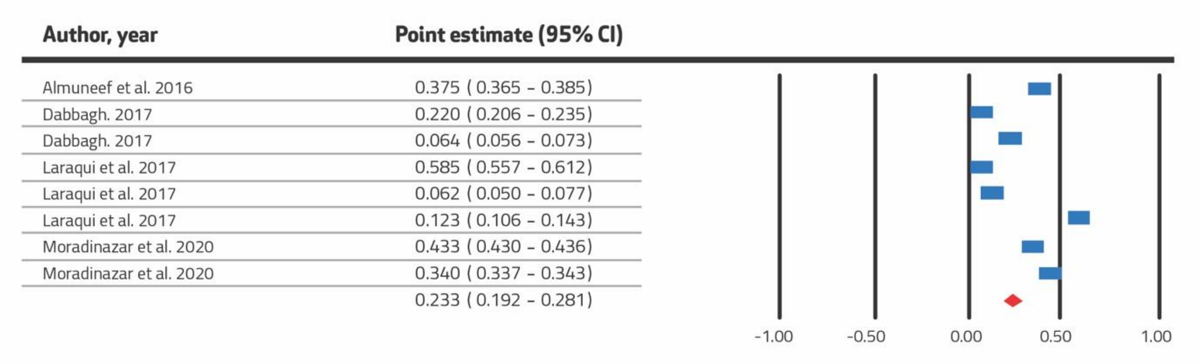

Appendix 16: Analyses for psychosis

Appendix 16.1: Forest plot for psychosis

Author, year	Point estimate (95% CI)
Abou-Saleh et al. 2001	0.007 (0.004 – 0.013)
Mohammadi et al. 2005	0.009 (0.008 – 0.010)
Mohammadi et al. 2005	0.002 (0.002 – 0.003)
Mohammadi et al. 2005	0.001 (0.001 – 0.001)
Mohammadi et al. 2005	0.000 (0.000 – 0.001)
Mohammadi et al. 2005	0.000 (0.000 – 0.001)
Mohammadi et al. 2005	0.005 (0.004 – 0.006)
Mufti et al. 2005	0.056 (0.044 – 0.070)
Ghanem et al. 2009	0.002 (0.001 – 0.003)
El-Wasify et al. 2011	0.006 (0.003 – 0.015)
El-Wasify et al. 2011	0.004 (0.001 – 0.011)
El-Wasify et al. 2011	0.001 (0.000 – 0.009)
El-Wasify et al. 2011	0.002 (0.001 – 0.010)
Ahmadvand et al. 2012	0.012 (0.008 – 0.018)
Ahmadvand et al. 2012	0.022 (0.012 – 0.039)
Ahmadvand et al. 2012	0.020 (0.011 – 0.036)
Nikfarjam et al. 2016	0.007 (0.005 – 0.009)
Veisani et al. 2017	0.045 (0.032 – 0.062)
	0.005 (0.003 – 0.009)

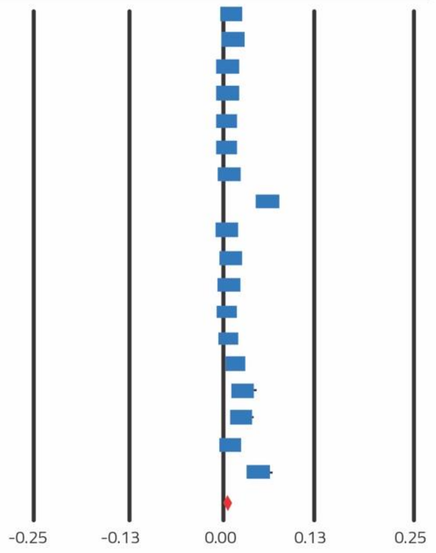

Appendix 16.2: Funnel plot for psychosis

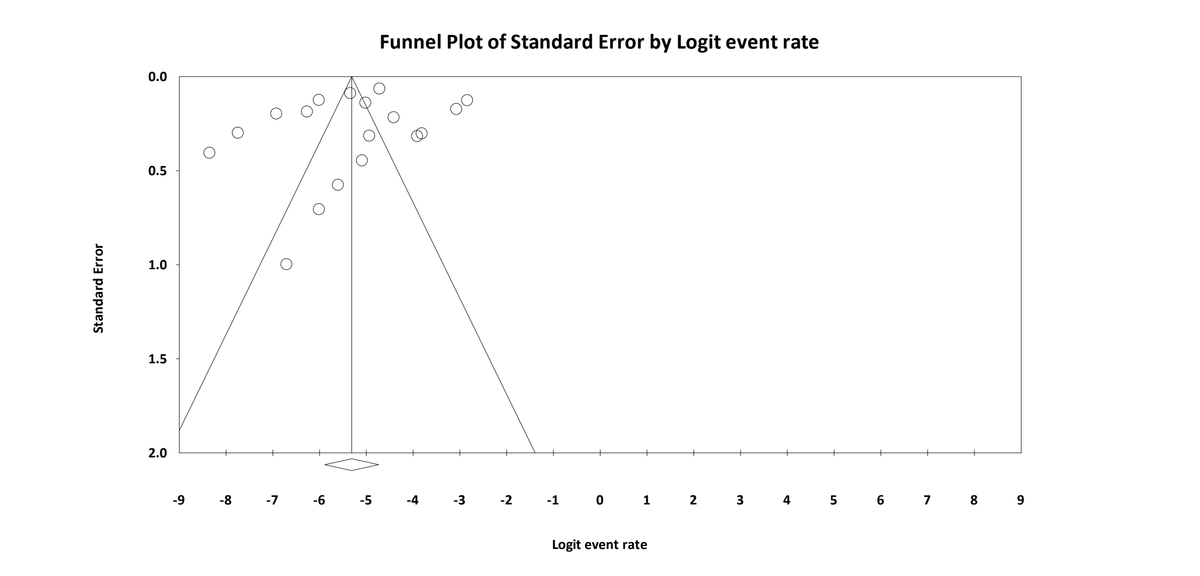

Appendix 16.3: Random-effects subgroup analysis of prevalence of psychosis stratified by potential determinants and methodological factors

	Point estimates and 95% CI							
	**No. of cohorts**	**Prevalence (%)**	**Lower limit**	**Upper limit**	**I^2 (%)**	**Q-value**	**df (Q)**	***p*-value**
**EMR Countries**								
Afghanistan	1	5.6	0.8	30.8	0	7.06	3.00	0.07
Egypt	5	0.0	0.0	1.0	42.06			
Iran	11	0.0	0.0	1.0	97.96			
UAE	1	1.0	0.0	6.0				
**Type of screened method**								
Diagnostic	17	0.5	0.3	0.9	98.05	0.06	1.00	0.80
Non-diagnostic	1	1.0	0.0	7.0	0.00			
**Country's income group**								
High	1	1.0	0.0	6.0	0.00	7.06	3.00	0.07
Low	1	5.6	0.0	0.8	30.8			
Lower-middle	5	0.0	0.0	1.0	42.06			
Upper-middle	11	0.0	0.0	1.0	97.96			

Appendix 16.4: Meta-regression for psychosis by mean-age and GDP

	**Coefficient**	**Standard Error**	**95% Lower limit**	**95% Upper limit**	**Z-value**	***p*-value**
**Model 1**						
Intercept	-0.54	4.85	-10.04	8.96	-0.11	0.91
Age	-0.12	0.13	-0.38	0.15	-0.88	0.38
**Model 2**						
Intercept	-4.45	0.93	-6.28	-2.62	-4.77	<0.01
GDP	< 0.0	0.0	< 0.0	0.0	-0.99	0.32

Appendix 17: Quality assessment for prevalence studies included in the meta-analysis

Authors, year	Sample frame appropriate	Sampling method appropriate	Sample size adequate	Details of sample and setting	Data analysis with sufficient coverage	Valid methods of identification	Condition measurements reliable	Appropriate statistics	Response rate adequate	Total Quality Score (%)
El-Sherbiny et al., 2016	1	1	1	1	1	1	0	1	1	8 (89%)
Almuneef et al., 2016	1	0	1	1	1	1	1	1	0	7(78%)
Baniasadi et al., 2019	1	1	1	1	1	1	1	1	0	8 (89%)
Gammouh et al., 2015	1	1	1	1	0	1	0	1	1	7 (78%)
Aly et al., 2018	1	1	1	1	1	1	0	1	0	7 (78%)
Tayefi et al., 2017	1	1	1	1	0	1	0	1	1	7 (78%)
Alvi et al., 2017	1	1	1	0	0	1	0	1	0	5 (56%)
Azizi et al., 2019	1	1	1	1	0	1	0	1	0	6 (67%)
Obeid et al., 2020	1	1	1	1	1	0	1	1	1	8 (89%)
Hamdan-Mansour et al., 2017	1	1	1	1	0	0	0	0	0	4 (44%)
Aoun et al., 2018	1	1	1	1	0	1	0	1	0	6 (67%)
Al Rashed et al., 2019	0	0	1	0	0	1	1	1	1	5 (56%)
Farhood et al., 2016	1	1	1	0	1	1	1	1	0	7 (78%)
Alhassan et al., 2018	0	0	1	0	0	1	0	0	0	2 (22%)
Rafiey et al., 2019	1	1	1	1	0	1	1	1	0	7 (78%)
Salah et al., 2015	1	1	1	1	0	1	1	1	1	8 (89%)
El-Gilany et al., 2018	1	1	1	1	1	1	0	1	1	8 (89%)
Bakhtiari et al., 2018	1	1	1	1	1	1	0	1	1	8 (89%)
Abou-Saleh et al., 2001	1	1	1	1	0	1	1	1	0	7 (78%)
Mohammadi et al., 2005	1	1	1	1	1	1	1	1	0	8 (89%)
Kim et al., 2007	1	1	1	1	0	1	1	1	1	8 (89%)

De Jong et al., 2003	1	1	1	0	0	0	1	1	1	6 (67%)
Bhamani et al., 2013	1	1	1	1	0	1	1	1	1	8 (89%)
Ghanem et al., 2009	1	1	1	1	0	1	1	1	1	8 (89%)
Roberts et al., 2009	1	1	1	1	0	1	1	1	1	8 (89%)
Kadri et al., 2007	1	1	1	1	0	1	1	1	1	8 (89%)
Nisar et al., 2004	1	1	1	0	0	0	1	1	0	5 (56%)
Mufti et al., 2005	1	1	1	0	0	0	1	0	0	4 (44%)
El-Wasify et al., 2011	1	1	1	1	1	0	1	1	1	8 (89%)
Alhasnawi et al., 2009	1	1	1	0	0	0	0	1	1	5 (56%)
Ahmadvand et al., 2012	1	1	1	1	0	1	1	1	0	7 (78%)
Kadri et al., 2010	1	1	1	1	1	1	1	0	1	8 (89%)
Scholte et al., 2004	1	1	1	1	1	0	1	1	0	7 (78%)
Karam et al., 2016	1	1	1	1	1	1	1	1	0	8 (89%)
Ruscio et al., 2017	1	1	1	0	1	1	1	1	0	7 (78%)
Mehrabi et al., 2019	1	1	1	1	1	0	0	1	1	7 ((78%)
Khaled et al., 2020	1	1	1	1	1	1	1	1	1	9 (100%)
Hasan et al., 2019	1	1	1	0	1	0	0	1	1	6 (67%)
Khazaie et al. 2019	1	1	1	1	1	1	1	1	0	8 (89%)
Degenhardt et al., 2019	1	1	1	0	0	0	1	1	0	5 (56%)
Khaled et al., 2019	1	1	1	1	1	0	0	1	1	7 (78%)
Kausar et al., 2015	1	1	1	0	0	0	1	1	1	6 (67%)
Noorbala et al., 2015	1	1	1	1	1	1	1	0	1	8 (89%)
Sharifi et al., 2017	1	1	1	1	0	1	1	0	0	6 (67%)
Stubbs et al., 2017	1	1	1	1	1	0	0	1	0	6 (67%)
Rafiey et al., 2017	1	0	1	1	1	0	0	1	0	5 (56%)
Laraqui et al., 2017	0	1	1	1	0	0	0	1	0	4 (44%)
Dabbagh, 2017	1	1	1	1	1	0	0	1	1	7 (78%)
Roshanpajouh et al., 2019	1	1	1	1	1	0	1	1	1	8 (89%)

Nikfarjam et al., 2016	1	1	1	1	1	0	0	1	0	6 (67%)
Stubbs et al., 2016	0	1	1	1	1	1	1	1	1	8 (89%)
Veisani et al., 2017	1	1	1	1	1	1	1	1	0	8 (89%)
Moradinazar et al., 2020	1	0	1	1	1	0	0	1	0	5 (56%)
Asokan et al., 2019	1	1	1	1	1	1	1	1	1	9 (100%)

Note: The Joanna Briggs Institute (JBI) Critical Appraisal Checklist consists of the following items: representativeness of the sampling frame, appropriateness of the sampling method, appropriateness of sample size, adequateness of details of the sample and setting, sufficient analysis of data coverage, validity and reliability of the measures, appropriateness of reported statistics and the adequateness of response rate.

Each item either received a ‘yes’ - indicating a low risk for bias, ‘no’ - indicating a high risk of bias, and ‘unclear’ - indicating uncertainty over potential biases. These were further translated into scores, with ‘yes’ being scored as 1 and ‘no’ and ‘unclear’ being scored as 0. Intervals for scores were arbitrarily decided and total scores ≤ 4 were rated ‘low-quality’, between 5-7 (inclusive) were rated ‘medium-quality’ and scores ≥ 8 were rated ‘high-quality’. Studies with high risk of biases (or low-quality studies) were not excluded.

Appendix 18: Trends across country-level factors, age and population

Country-level factors

Across EMR countries, there was no evidence of a difference between disorder prevalence rates except for OCD, phobic and panic disorders and substance-use, with Morocco consistently reporting one of the highest prevalence estimates for each. An additional trend observed across countries was that low-income countries had significantly higher rates of PTSD and phobic disorders. Similarly, country's GDP was found to have a negative association with PTSD and phobic disorders.

We also investigated the association between mental disorders and a novel country-level factor in the region, namely, regime type. Hybrid regimes (a regime that has not fully transitioned from an authoritarian to a democratic one, for example, Tunisia, Pakistan, Lebanon, Morocco) were found to have significantly higher prevalence of phobic and panic disorders. Additionally, substance use was significantly higher in hybrid regimes. This links with the review's finding of Pakistan, a hybrid regime, having the highest prevalence of substance use in the region.

Age

Mean-age of participants was found to have an association with substance use in this review, with older people having higher rates of substance use. Overall, the non-fatal burden of mental disorders in the EMR is found to be highest between the ages of 25 – 49 years, but there were no other age-related trends observed across the specific disorders in this review. There is a need for a more detailed investigation of the prevalence of mental disorders across the lifespan to identify possible trends that could have been missed by this review.

Trauma-exposed populations

Depressive disorders, GAD and PTSD were higher among trauma exposed populations. Although there was only evidence for a significant difference of higher PTSD and a marginally significant difference of higher GAD among this population group (as compared to the general population), this trend has been well-established by previous research by Baxter and colleagues (2013) as well as more recently by Charlson and colleagues (2019), for both

depressive and types of anxiety disorders Generally, there was a lack of evidence for differences between these two population groups, and this was mainly due to an insufficient number of studies on trauma-exposed populations.

References:

Baxter AJ, Scott KM, Vos T, Whiteford HA. Global prevalence of anxiety disorders: a systematic review and meta-regression. Psychological medicine. 2013 May 1;43(5):897.

Charlson F, van Ommeren M, Flaxman A, Cornett J, Whiteford H, Saxena S. New WHO prevalence estimates of mental disorders in conflict settings: a systematic review and meta-analysis. The Lancet. 2019 Jul 20;394(10194):240-8.
